# First evidence of *Proganochelys quenstedtii* (Testudinata) from the *Plateosaurus* bonebeds (Norian, Late Triassic) of Frick, Canton Aargau, Switzerland

**DOI:** 10.1186/s13358-022-00260-4

**Published:** 2022-10-27

**Authors:** Torsten M. Scheyer, Nicole Klein, Serjoscha W. Evers, Anna-Katharina Mautner, Ben Pabst

**Affiliations:** 1 Palaeontological Institute and Museum, University of Zurich, Karl-Schmid-Strasse 4, 8006 Zurich, Switzerland; 2 Institute of Geosciences, Paleontology, University of Bonn, Nussallee 8, 53115 Bonn, Germany; 3 Department of Geosciences, University of Fribourg, Ch. du Musée 6, 1700 Fribourg, Switzerland; 4 Sauriermuseum Aathal, Zürichstrasse 69, 8607 Aathal-Seegräben, Switzerland

**Keywords:** Testudines, Klettgau Formation, Gruhalde Member, Turtle shell, Brain endocast, Bony labyrinth

## Abstract

*Proganochelys quenstedtii* represents the best-known stem turtle from the Late Triassic, with gross anatomical and internal descriptions of the shell, postcranial bones and skull based on several well-preserved specimens from Central European fossil locations. We here report on the first specimen of *P. quenstedtii* from the Late Triassic (Klettgau Formation) Frickberg near the town of Frick, Canton Aargau, Switzerland. Similar to other Late Triassic ‘*Plateosaurus*-bearing bonebeds’, *Proganochelys* is considered to be a rare faunal element in the Swiss locality of Frick as well. The specimen, which is largely complete but was found only partially articulated and mixed with large *Plateosaurus* bones, overall resembles the morphology of the classical specimens from Germany. Despite being disarticulated, most skull bones could be identified and micro-computed tomography (CT) scanning of the posterior skull region reveals new insights into the braincase and neurovascular anatomy, as well as the inner ear region. These include the presence of a fenestra perilymphatica, potentially elongated cochlear ducts, and intense vascularization of small tubercles on the posterior end of the skull roof, which we interpret as horn cores. Other aspects of the skull in the braincase region, such as the presence or absence of a supratemporal remain ambiguous due to the fusion of individual bones and thus lack of visible sutures (externally and internally). Based on the size of the shell and fusion of individual elements, the specimen is interpreted as a skeletally mature animal.

**Supplementary Information:**

The online version contains supplementary material available at 10.1186/s13358-022-00260-4.

## Introduction

The genus *Proganochelys* Baur, 1887 from the Late Triassic (Norian), represents one of the best known and most widely distributed members of the evolutionary turtle stem (Baur, 1887; Gaffney, 1990), showing a Pangaean wide distribution with fossils being recorded from Germany, Thailand, and potentially Greenland (Joyce, 2017; Marzola, 2019). Together with fossils of the genus *Proterochersis* from the Late Triassic (Middle Norian) of Germany and Poland and *Palaeochersis* and *Waluchelys* (Sterli et al., 2021) from the Late Triassic of Argentina, *Proganochelys* represents the critical stage in turtle evolution, in which the iconic rigid shell consisting of a dorsal carapace and ventral plastron connected by a bony bridge appeared for the first time (e.g., Fraas, 1899, 1913; Joyce, 2017; Rougier et al., 1995; Sterli et al., 2007, 2021; Szczygielski & Sulej, 2016, 2019; Zangerl, 1969). Prior to this stage in evolution, stem turtles, such as *Odontochelys semitestacea* and *Eorhynchochelys sinensis* from the Late Triassic (Carnian) of China (Li et al., 2018; Li et al., 2008, see also Lyson et al., 2013), and *Pappochelys rosinae* from the Middle Triassic (Ladinian) of Germany (Schoch & Sues, 2015, 2018), lacked a rigid shell. Instead, these taxa presented different precursory, non-fused semi-rigid stages in proto-shell formation.

The genus *Proganochelys* comprises currently two species (Joyce, 2017): *P. quenstedtii* from Europe (Gaffney, 1990) and *P. ruchae* from Thailand (Broin, 1984). Additional associated material from a single but fragmentary specimen from the Flaming Fjord Formation at Carlsberg Fjord, Sermersooq, E Greenland was tentatively identified as cf. *Proganochelys* sp. (Jenkins et al., 1994), but with new stem-turtle material being described, this assignment appears not to be valid or at least even more tentative than previously anticipated (Marzola, 2019). As such, *P. quenstedtii* is the only well-known species as it is represented by several complete and articulated or partially articulated specimens and most anatomical and palaeobiological studies on *Proganochelys* are thus centred around the European fossils (e.g., Ballerstedt, 1921; Gaffney & Meeker, 1983; Gaffney, 1983a, 1983b, 1985, 1990; Jaekel, 1916; Joyce, 2017; Joyce & Gauthier, 2004; Kordikova, 2002; Lautenschlager et al., 2018; Scheyer & Sander, 2007; Werneburg, 2015, 2021; Werneburg et al., 2015).

Due to the depositional environment and limb morphology, *Proganochelys* was initially interpreted as a fresh water inhabitant and bottom walker, which was not exclusively aquatic or terrestrial (Gaffney, 1990: p.5). On the other hand, *Proganochelys* is mostly found in taphocoenoses with plateosaurid sauropodomorphs, such as *Plateosaurus trossingensis* (e.g., Jaekel, 1916; Schoch, 2011) and *Issi saaneq* (Beccari et al., 2021; Jenkins et al., 1994). These dinosaurs are only known from continental sediments and notably lacking from aquatic assemblages (Joyce, 2017; Joyce et al., 2009). In addition, *Proganochelys* shell bone histology is comparable to that of extant tortoises (Scheyer & Sander, 2007) which, together with the presence of osteoderms, a tail club, and short limbs with strong claws argued in favour of a terrestrial life style similar to that of modern tortoises (Joyce & Gauthier, 2004).

Here we report on a new specimen of *Proganochelys* from the Norian *Plateosaurus-*bearing Klettgau Formation of Frick in Canton Aargau, Switzerland, thus expanding the known distribution of the taxon to include this major European dinosaur fossil Lagerstätte. Furthermore, we present novel data on the cranial anatomy of the specimen, including micro-computed tomography scan data of the braincase and inner ear region.

## Materials and methods

**Fig. 1:**
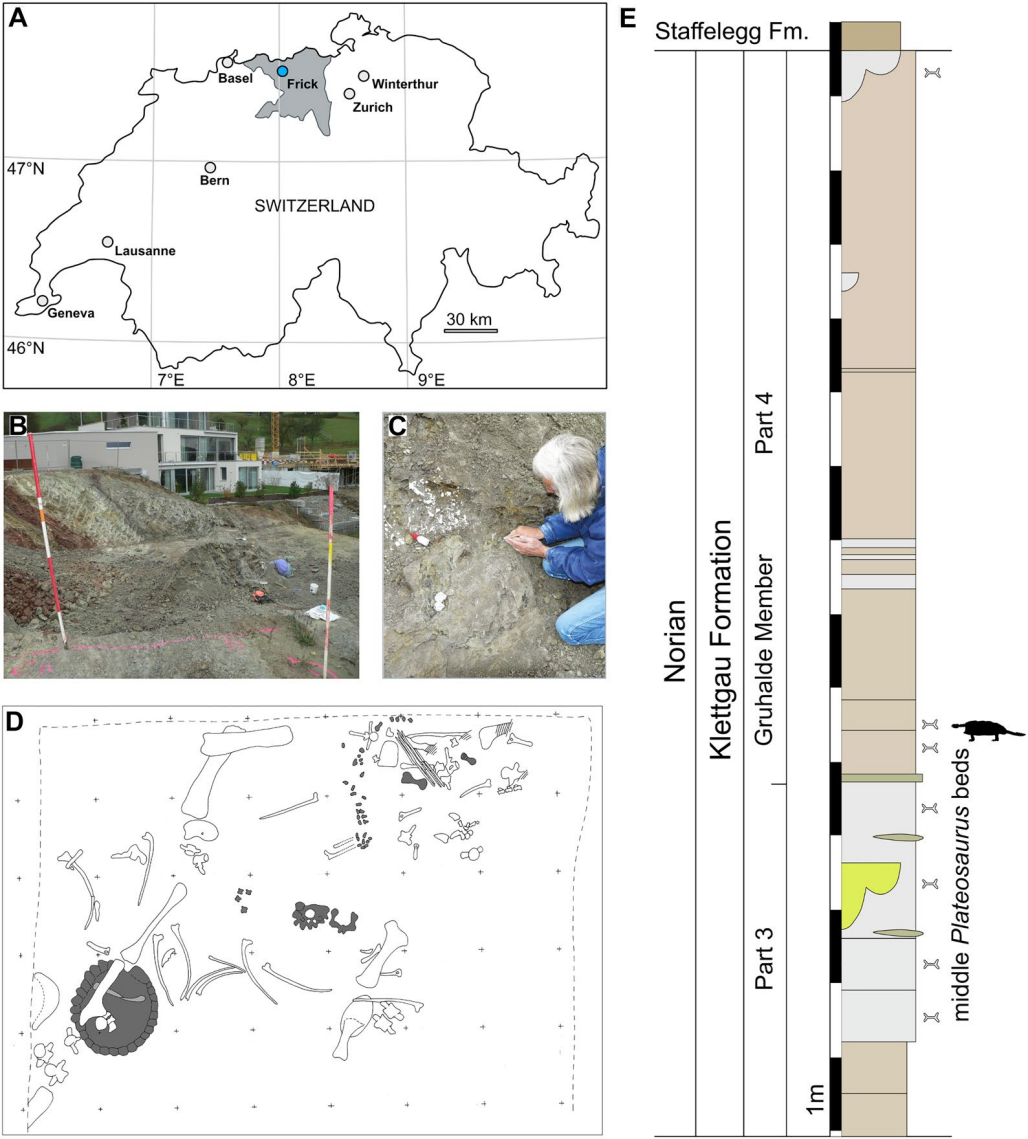
Geographical and geological overview of the find of *Proganochelys quenstedtii* (SMF 09-F2). **A,** Schematic overview map of Switzerland with the town of Frick in Canton Aargau (in grey) highlighted [redrawn from 3D map viewer at https://www.swisstopo.admin.ch/]. **B,** Photograph of the construction site where the sediments of the Gruhalde Member are exposed at Frickberg (Image courtesy: Grabungsteam Frick, used with permission). **C,** Excavation of SMF 09-F2 by one of us (BP; Image courtesy: Grabungsteam Frick, used with permission). **D,** Schematic map of the excavation site using a 50 × 50 cm grid (indicated by the small + signs). The bones belonging to SMF 09-F2 are highlighted in dark grey. **E,** Simplified sedimentological column based on the exposed profile (based on Jordan et al., 2016 in Tschopp et al., 2020) in the clay pit Gruhalde and the finding layer of SMF 09-F2 at Frickberg indicated

The new specimen, SMF 09-F2, was found in 2009 in the course of a housing construction project at Frickberg, a hill opposite the famous *Plateosaurus*-bearing clay pit Gruhalde in the town of Frick, northern Switzerland (Hofmann & Sander, 2014; Sander, 1992). The specimen was found scattered and mixed with large disarticulated *Plateosaurus* bones over an area about 3 times 3 m, but with the shell still articulated (Fig. 1A–D).

The sediments exposed in the construction site belong to the upper part (part 4—‘Obere Bunte Mergel’ or upper variegated marls) of the Gruhalde Member of the Klettgau Formation, which are Norian in age (Fig. 1E). These marls are equivalent in stratigraphy, lithology and clay mineralogy to the Norian Trossingen Formation (‘Knollenmergel’ or ‘Feuerletten’ beds) in South and Central Germany (e.g., Matter et al., 1988; Sander, 1992), and eastern France (Weishampel & Westphal, 1986). The same layers at the clay pit Gruhalde in Frick are dominated by the presence of the sauropodomorph *Plateosaurus*, including dozens of well-preserved articulated or semi-articulated skeletons (e.g., Hofmann & Sander, 2014; Klein & Sander, 2007; Nau et al., 2020; Sander, 1992), as well as few remains of theropod dinosaurs, such as the recently described neotheropod *Notatesseraeraptor frickensis* (Oettl-Rieser & Zahner, 2018; Zahner & Brinkmann, 2019).

SMF 09-F2 is housed in the collection at the “Sauriermuseum Frick”, at Frick in Canton Aargau, Switzerland. It consists of a largely complete carapace and parts of the plastron, associated with a fragmentary skull, cervical and caudal vertebrae, a left humerus and ulna found close to additional phalanges including two unguals and additional limb osteoderms, the lower right hind limb including the autopodium (lacking the femur), a complete pelvis still in articulation and a fragmented postpelvic hypoischium. Due to the find situation (Fig. 1D), the size of the preserved plates and bones (with no duplicates), all elements are considered to belong to the same individual. In the field, the carapace was found separated from the other elements belonging to the specimen that were spread diagonally across the construction site and mixed with large *Plateosaurus* bones (Fig. 1D). The skull bones were found in association over a limited area of a few square centimetres, but not articulated with each other anymore.

Mechanical preparation of the fossil was done by one of us (BP), who also led the excavation. Missing parts of the carapace, especially on the left side, were mirrored and modelled for display purposes, based on preserved parts from the right side. The carapace has been found in visceral view and preparation was done from the inside first. Later the carapace was mounted on a bed of synthetic supporting material to prepare the outside of the carapace as well. Due to the extremely thin and fragile nature of the shell, it cannot be easily taken off or flipped over without risking breakage. The description of the internal morphology is, therefore, limited and based largely on photographs that have been taken during the process of preparation. See Tables 1 and 2 for measurements.

**Table 1:** Measurements of SMF 09-F2 skull bones. Measurements in [mm]

Element	Length	Width/Height
Left maxilla	50.4	31.7 (height)
Left prefrontal	50.4	18.5 (height)
Left quadratojugal	23.8	32.5 (height)
Left quadrate	23.1	36.8 (height)
Frontals	35.5	35.8 (width)
Braincase fragment	26.7	65.9 (maximum width) 46.2 (minimum width) 54.1 (height)

**Table 2:** Measurements of SMF 09-F2 postcranial elements. Measurements in [mm]

Element	Length	Width
Cervical	19.3	87.5 (as reconstructed)
Vertebral 1	146.2	278.0
Vertebral 2	118.6	295.0
Vertebral 3	89.9	265.0
Vertebral 4	108.1	221.0
Supracaudal	29.5	159.0 (as reconstructed)
Pleural 1 (right)	92.34	131.5
Pleural 2 (right)	132.26 (as preserved)	163.5
Pleural 3 (right)	129.35 (as preserved)	147.6
Pleural 4 (right)	96.9	114.1
Humerus Humerus mid-shaft	156.3	85.5 proximal 64.75 distal 22.1 × 16.8
Ulna Ulna mid-shaft	100.1	39.5 proximal 32.1 distal (as preserved) 17.6 maximum width
Metatarsal left autopod	26.5	20.2 (proximal) 18.3 (distal)
Tibia	96.1	51.7 (proximal expansion) 20.0 (dorsoventral thickness) 17.5 (shaft diameter) dorsoventral thickness shaft 8.1 distal expansion 30.2 dorsoventral thickness 12.6 mm
Fibula	92.2 length as preserved (approx. reconstructed 101)	23.9 proximal expansion 8.3 thickness 13.0 shaft diameter 6.3 max. shaft width 26.2 distal expansion 5.2 thickness
Metatarsalia right autopod	Mt V length 13.5 mt IV length 35.1 mt III length 39.8 mt II length 33.1 mt I length 17.8	
Astragalus right autopod	26.6 length	approximately 33.8 mm width
Calcaneum right autopod	15.6 length	approximately 13.5 mm width
phalanges of the right pes	ph 5 length 8.1 ph 4 length 12.5 ph 3 length 16.6 ph 2 length 15.9 ph 1 length 12.5	
unguals of the right pes	un 5 length 22.2 un 4 length 22.6 un 3 length 30.3 un 2 length 28.7 un 1 length 24.4	
Isolated phalanx left autopod	16.8 length	15.5 greatest width
Phalanx articulated with ungual left autopod	24 length 32.2 length	14.8 greatest width 15.9 greatest width
Isolated ungual left autopod	23 length	13 greatest width
Sesamoid left autopod	15 diameter	

The large posterior skull fragment of SMF 09-F2 was scanned using a NIKON XTH 225 ST CT Scanner housed at the Anthropological Department of the University of Zurich. The micro-computed tomography scan was taken with a voltage of 91 kV and a current of 345 µA, yielding a voxel size of 0.03758 mm, with no filter used. Reconstruction of the virtual model was performed using Mimics 23.0. CT scans and derivative 3D models are deposited on the online repository MorphoSource (see Declarations section).

For comparison, a scan of *Proganochelys* skull SMNS 16890 (original CT scan data set from Werneburg et al., 2015 which was also shown in Lautenschlager et al., 2018) was used to generate a 3D model in Mimics as well. After exporting the surface model, it was imported into Meshlab v.2022.02 (Cignoni et al., 2008) and rendered using the “Electronic microscope” shader.

Museum Abbreviations: **MB**, Naturhistorisches Museum Berlin, Germany; **SMF**, Sauriermuseum Frick, Canton Aargau, Switzerland; **SMNS**, Staatliches Museum für Naturkunde Stuttgart, Germany.

## Systematic palaeontology

Testudinata Klein, 1760 [(Joyce et al., 2020)].

*Proganochelys* Baur, 1887

*Proganochelys quenstedtii* Baur, 1887

Remarks—The cranial and postcranial anatomy of SMF 09-F2 is largely congruent with the in-depth description provided previously for *Proganochelys quenstedtii* (Gaffney, 1990), based mainly on well-preserved specimens from Trossingen and Halberstadt in Germany. The Swiss turtle has only a fragmentary plastron, which does not show any extragular projections, the orientation of which is still currently the only character separating the abundant *P. quenstedtii* material from the very few and heavily fragmented specimens of *P. ruchae* from Thailand (Broin, 1984). Given the aforementioned congruence of all other anatomical aspects, however, and given the geographic proximity with the abundant German material, we refer SMF 09-F2 to *Proganochelys quenstedtii* herein.

## Results

### Skull

**Fig. 2:**
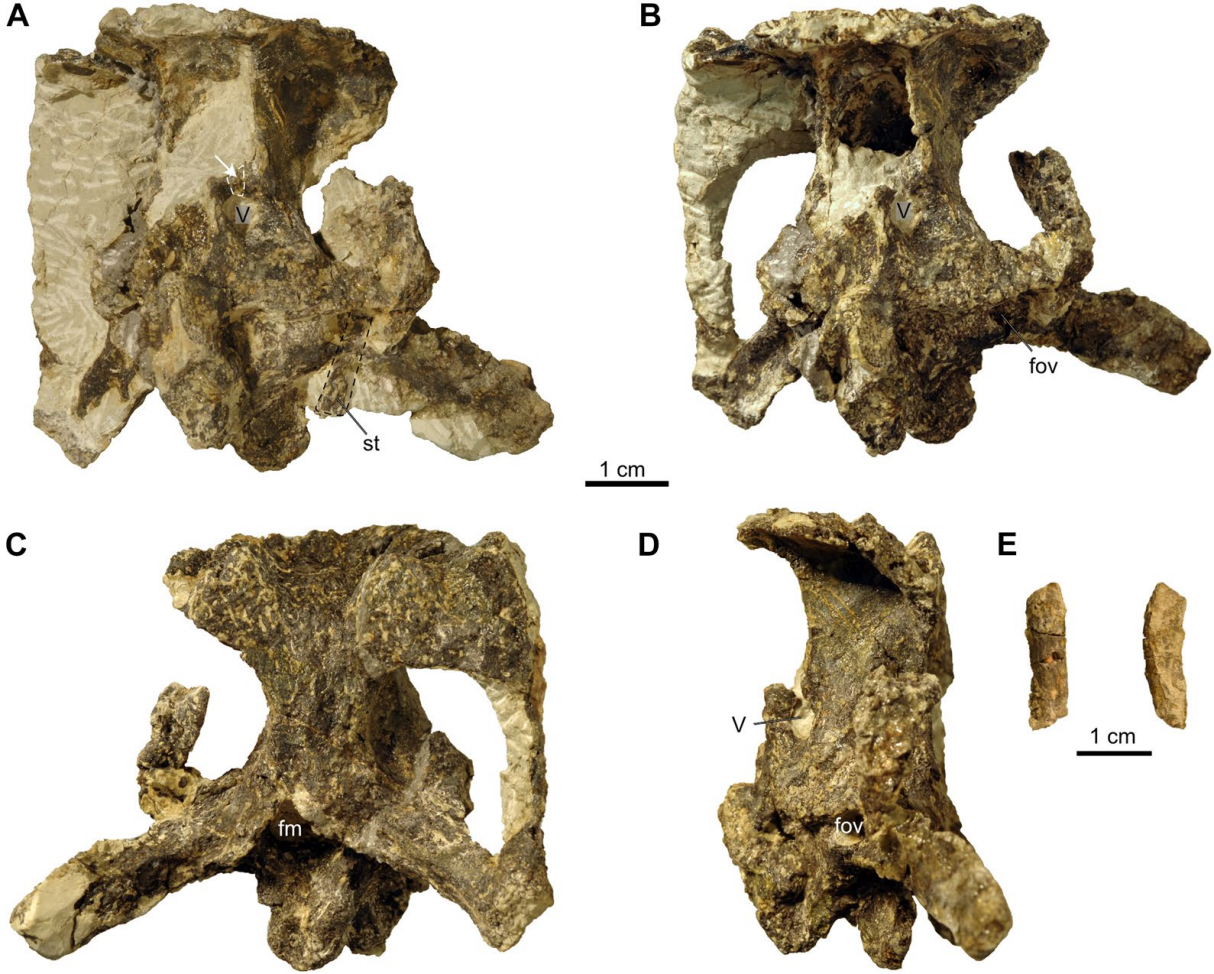
Braincase of *Proganochelys quenstedtii* (SMF 09-F2). **A,** Braincase during preparation in anterior view. Note that at this stage the prootic foramen for the trigeminal nerve was complete and the shaft of the stapes was still articulated with the braincase close to the fenestra ovalis (the small fragment indicated by the white stippled line and arrow as well as the shaft of the stapes are kept separately with the braincase). **B,** Braincase after preparation in anterior view. **C,** In posterior view. **D,** In left lateral view. Abbreviations: fm, foramen magnum; fov, fenestra ovalis; st, stapes; V, prootic foramen for the trigeminal (CN V) nerve

Of the skull, the posterior region of the skull (Fig. 2; described in a separate section below) including the parietals, supratemporals, the median-most part of the left postorbital, the right squamosal, and the braincase elements containing the supraoccipital, the opisthotics, exoccipitals, prootics, as well as the basioccipital and basisphenoid are preserved in articulation. These skull parts are compressed in overall mediolateral direction and the bones have a fragile appearance, showing numerous tiny cracks and sediment-filled veins. Sutures are not well visible externally because of poor preservation and even internally, using the CT data, individual bone boundaries are difficult to discern. This makes it difficult to comment in detail on previously proposed suture lines and skull reconstructions (e.g., Gaffney, 1990), but we include such statements whenever possible and relevant.

As indicated by photographs taken during and after preparation, slight damage to this skull part is noticeable (Fig. 2A–D). A tiny thin bone part, as well as an elongated rod-shaped and slightly bent bone part of 2 cm length appear to be the upper rim of the prootic foramen for the trigeminal (CN V) nerve and the shaft of the left stapes, respectively. The removed sediment infilling of the braincase, as well as the small bone parts are now kept separately with the braincase.

**Fig. 3:**
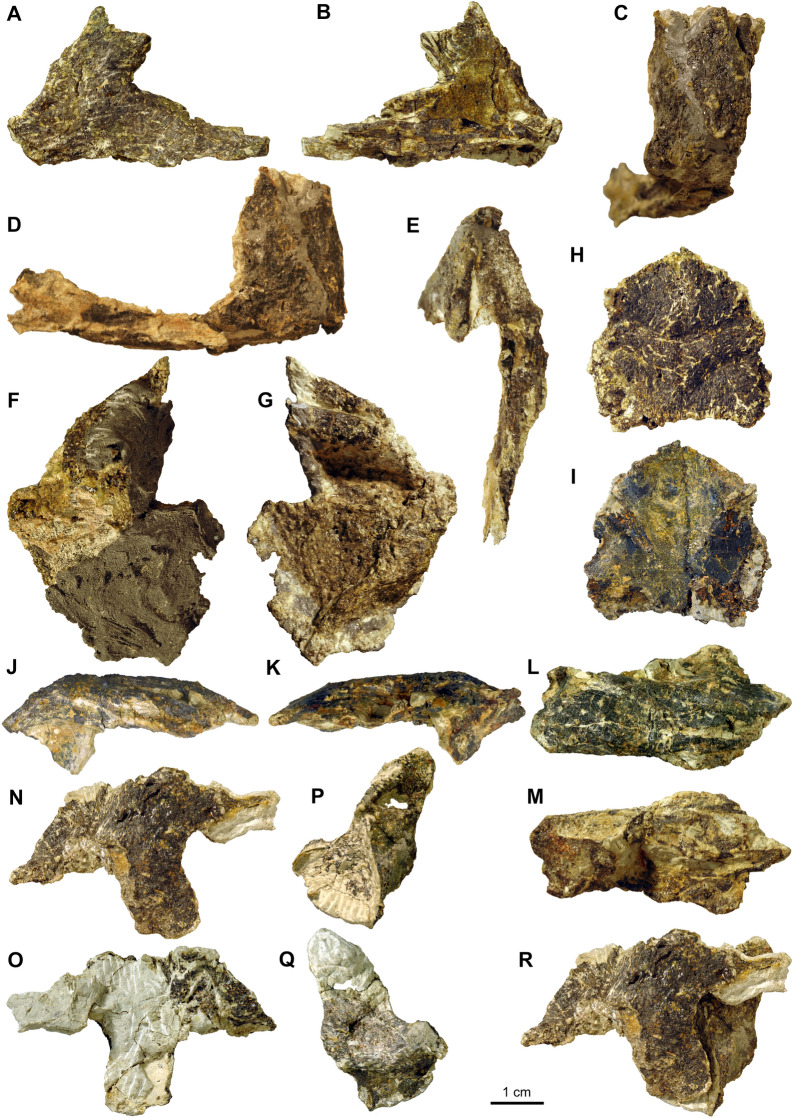
Skull elements of *Proganochelys quenstedtii* (SMF 09-F2). **A, B,** Left maxilla (A lateral, B medial view). **C–E,** Right maxilla, premaxillae and part of jugal (C anterior, D right lateral, E ventral view); **F, G,** Nasals and possible parts of right prefrontal (F dorsal, G ventral view). **H, I,** Frontals (H dorsal, I ventral view); **J–M,** Left prefrontal and postorbital (J lateral, K medial, L dorsal, M ventral view); **N, O,** Left quadratojugal and part of jugal (N lateral, O medial view); **P, Q,** Left quadrate (P lateral, Q medial view); **R,** Reassembled left quadratojugal and quadrate in lateral view

In addition, the frontals, the left quadratojugal with part of the remaining left jugal, the left quadrate, the left prefrontal and dorsal part of postorbital the left maxilla, the nasals potentially also with part of the right prefrontal, and the premaxillae still articulated with the right maxilla and anterior process of the right jugal were found isolated and scattered over a small area. The bones can be roughly rearticulated except the maxillae and premaxillae (Additional file 1: Fig. 1A, B; Additional file 2: Fig. 2A–H), indicating that from the left skull portion only large parts of the postorbital and jugal are missing. No elements of the lower jaw could be recovered. All these bones basically conform to previous descriptions of *Proganochelys quenstedtii* (Gaffney, 1990), but due to their isolated nature (Fig. 3), a brief description is given below.

The part comprising the premaxillae still articulating with the right maxilla (Fig. 3A–E) is strongly crushed. The posterior maxillary process (still in contact with the anterior process of the right jugal) has separated and is dislocated medially from the anterior ascending plate-like part of the maxilla. The premaxillae are strongly distorted, but the medial suture can be traced from the ventral margin along the medial ascending processes. The right external naris is still confined between the right maxilla and the right premaxilla. The left maxilla is complete externally but medially, parts of the bone close to the sutures with the prefrontal, jugal, and palatal elements are broken, revealing the internal microstructure of the maxilla here. Anteriorly, the lateral border of the apertura narium externa is visible as a smooth and slightly concave depression in the bone margin. Ventrally, the triturating surface is formed by labial and lingual sharp ridges framing a deep trough. The labial ridge, i.e., the lateral ventral margin of the maxilla is slightly bent inward, indicating that the element is slightly distorted labiolingually/mediolaterally.

The extremely fragile fragment that comprises the nasals and maybe also parts of the right prefrontal is heavily reconstructed dorsally (Fig. 3F, G); the ventral (internal) side, however, is better preserved and reveals two low bony ridges framing the flat bone surface, which are interpreted here as the parasagittal ridges encompassing the sulcus olfactorius (i.e., the olfactory tract). A conspicuous depression could indicate the connection of the right nasal with parts of the right prefrontal in this area.

The frontals are still articulated and overall well-preserved (Fig. 3H, I). The anterior and anterolateral margins of both elements, articulating with the nasals, is convex; the posterior margin contacting the parietals is slightly concave. The straight to slightly convex lateral margins contact the postorbitals. Dorsally, the bone surface of the frontals is rugose and shows the same scute pattern as was previously described in SMNS 16980 (Gaffney, 1990: p. 29, Fig. 17; two smaller scutes separating larger anterior and posterior scutes). Ventrally, the frontals have a smooth bone surface, each with a weak crista cranii.

The left prefrontal is overall quite three-dimensionally preserved, carrying a dorsal ornamental boss or bump, and being still articulated with the anterior part of the postorbital (Fig. 3J–M). The suture between both bones is not discernible and the medial margins of the bones are distorted. Anteroventrally, a descending process of the prefrontal is partly preserved.

Only a small and heavily distorted remnant of the left jugal appears to be still attached to a largely undistorted quadratojugal (Fig. 3N, O). The latter has a recurved shape in lateral view. Its shape confirms the borders of the bone indicated on SMNS 16980 (Gaffney, 1990: fig. 16). The anterior contact with the jugal is mostly straight. The posterior contact with the underlying quadrate (Fig. 3P–R) is straight ventrally and convex dorsally. The quadrate is a very fragile looking bone with most of the borders showing some damage and distortion. The articular surface with the quadratojugal is visible as a slightly concave triangular surface.

### Braincase and internal anatomy

**Fig. 4:**
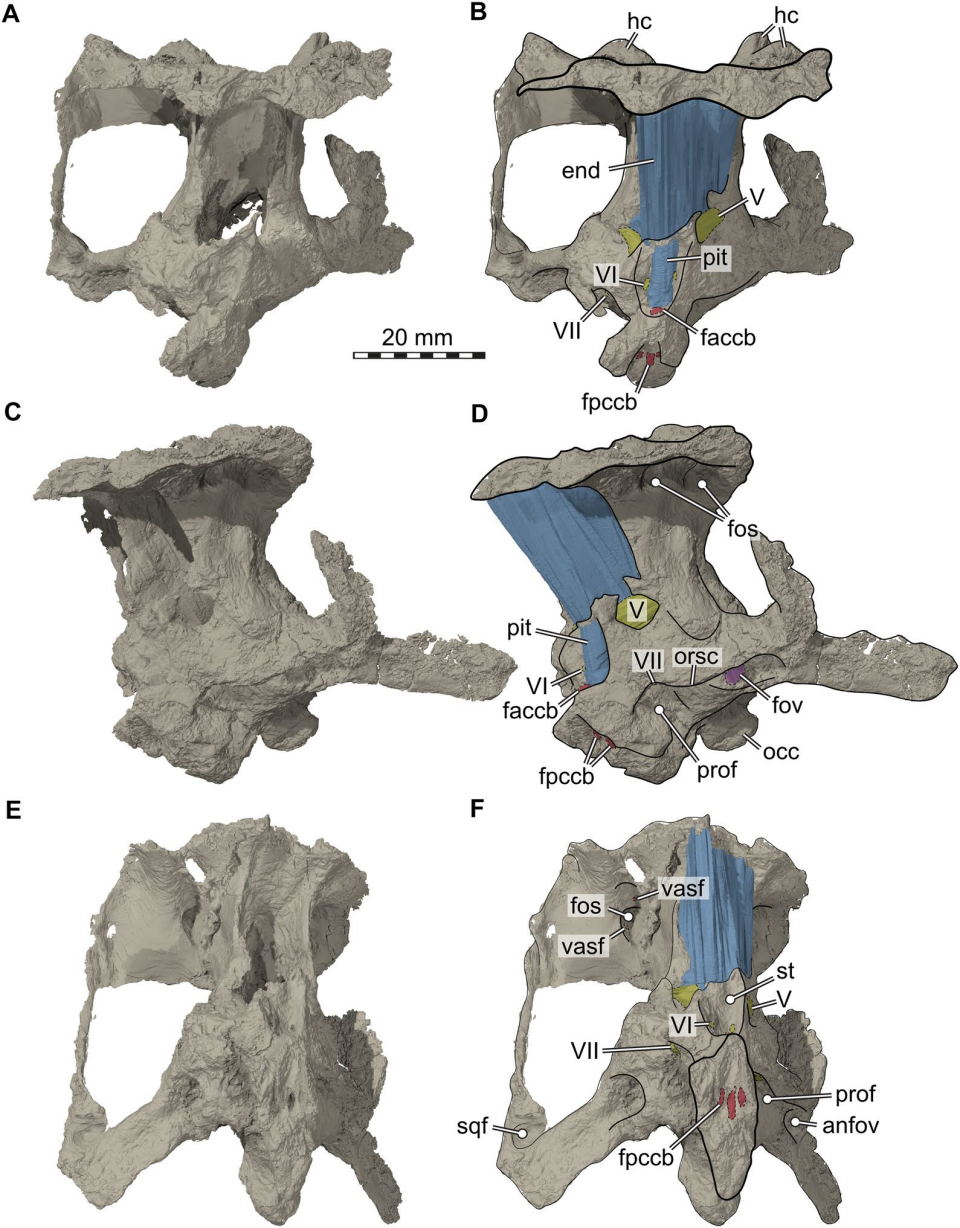
3D renderings of the braincase of *Proganochelys quenstedtii* (SMF 09-F2). **A,** Anterior view; **B,** Same as A, but with soft tissue endocasts and interpretative line drawings included; **C,** Left lateral view; **D,** Same as C, but with soft tissue endocasts and interpretative line drawings included; **E,** Anteroventral view; **F,** Same as E, but with soft tissue endocasts and interpretative line drawings included. Abbreviations: anfov, antrum to fenestra ovalis; end, cranial endocast; faccb, foramen anterius canals caroticus basiphenoidalis; fos, fossa; fov, fenestra ovalis; fpccb, foramen posterius canalis caroticus basisphenoidalis; hc, horn core; occ, occipital condyle; orsc, orbitosphenoidal crest; pit, endocast of pituitary fossa; prof, prootic fossa; sqf, squamosum fossa; st, sella turcica; V, prootic foramen for the trigeminal (CN V) nerve; VI, abducens nerve (CN VI) foramen; VII, facial nerve (CN VII) foramen; vasf, vascular foramen

**Fig. 5:**
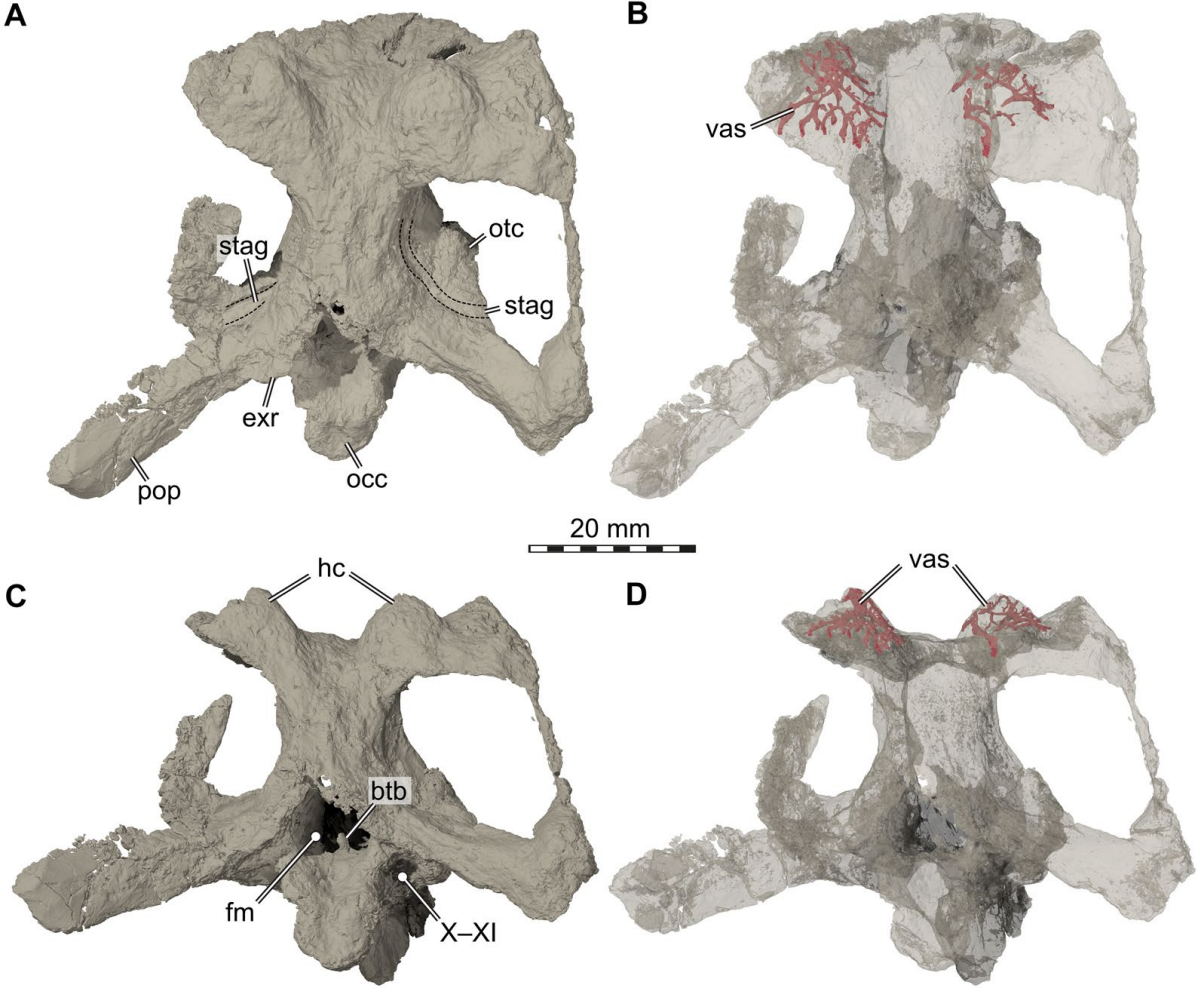
3D renderings of the braincase of *Proganochelys quenstedtii* (SMF 09-F2). **A,** Posterodorsal view; **B,** Same as A, but rendered transparently with horn core vascularization models rendered opaque; **C,** Posterior view; **D,** Same as C, but rendered transparent with horn core vascularization models rendered opaque. Note that dashed lines in A are margins of a weakly defined groove we interpret as the osteological correlate for the course of the stapedial artery. Abbreviations: btb, basis tuberculi basalis; exr, exoccipital ridge; fm, foramen magnum; hc, horn core; occ, occipital condyle; otc, otic capsule; pop, paroccipital condyle; stag, stapedial artery groove; vas, vascularization; X–XI, short canal for the CN X–XI (i.e., foramen jugulare intermedius of Sterli & Joyce, 2007)

**Fig. 6:**
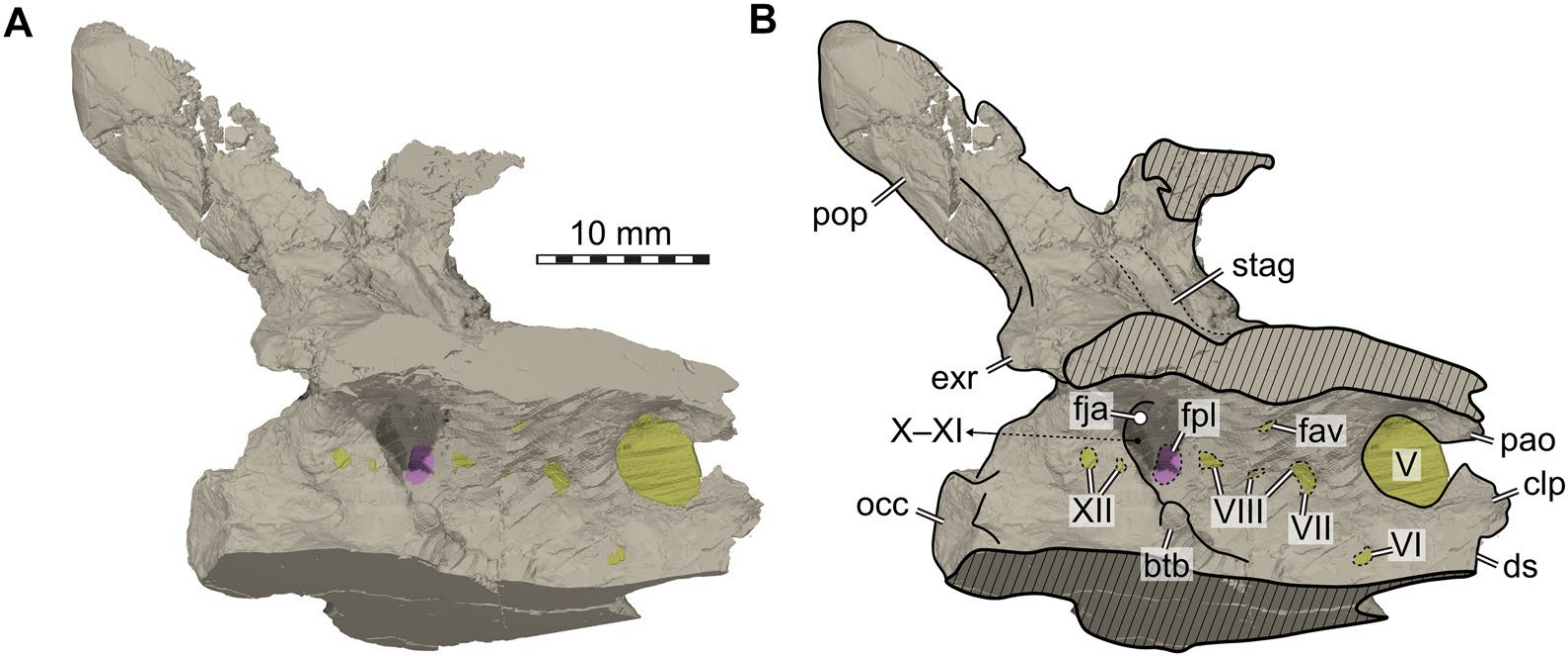
3D renderings of the sagittally and axially sectioned braincase of *Proganochelys quenstedtii* (SMF 09-F2). **A,** Dorsomedial view onto left side of sectioned braincase, exposing the medial surface of the braincase. Note that nerve endocasts and the endosseous labyrinth is also rendered. **B,** Same image as A, but with interpretative line drawings. Note that the arrow in B indicates the course of the CN X–XI from the foramen jugulare anterius through a short canal. Abbreviations: btb, basis tuberculi basalis; clp, clinoid process; ds, dorsum sellae; exr, exoccipital ridge; fav, foramen aquaducti vestibuli; foramen jugulare anterius (i.e., internal braincase opening for CN X–XI; fpl, fenestra perilymphatica; occ, occipital condyle; pao, pila antotica; pop, paroccipital process; stag, stapedial artery groove; V, prootic foramen for the trigeminal (CN V) nerve; VI, (internal) abducens nerve (CN VI) foramen; VII, (internal) facial nerve (CN VI) foramen; VIII, (internal) acoustic nerve foramina; X–XI, course of vagus (CN X) and accessory (CN XI) nerves; XII, hypoglossal nerve foramina

Although the braincase anatomy of SMF 09-F2 largely agrees with the previous description of *Proganochelys quenstedtii* (Gaffney, 1990), there are a few differences that we highlight here. The braincase (Figs. 4, 5, 6), including the supraoccipital, both parietals, both prootics, both opisthotics, both exoccipitals, the basisphenoid and the basioccipital, is nearly completely preserved. The parasphenoid part of the parabasisphenoid complex (Sterli et al., 2010) is damaged, as the cultriform process is broken off, as is the right clinoid process (Fig. 4A, B). Besides the actual braincase elements, the right squamosal is preserved in articulation with the fossil. Sutures are only observable as partial traces along the specimen as well as in CT slices, making a precise delimitation of most elements impossible. The material shows mediolateral as well as anteroposterior shearing of the dorsal vs. ventral braincase parts (Figs. 4, 5). There is considerable damage on the left skull side toward the position of the quadrate. In this area, a large piece of the braincase is oddly attached to the left otic capsule, and it seems that this may be part of a taphonomically deformed partial quadrate that is bend anterodorsally from its original position. Despite this, the fossil is in overall good 3D preservation, which allowed us to segment both endosseous labyrinths, a cranial endocast and the endocast of the pituitary fossa, as well as the canals of several cranial nerves.

Although the parietal–squamosal contact area is preserved on the right side of SMF 09-F2, it is unclear if a supratemporal is present. The presence of this bone was hypothesized for *Proganochelys quenstedtii* (Gaffney & Meeker, 1983; Gaffney, 1983b, 1990), and also reported for *Palaeochersis talampayensis* (Sterli et al., 2007). Some later specimen-based studies also identified the supratemporal in *P. quenstedtii* (Kordikova, 2002), and overview studies generally followed the interpretation that a supratemporal is present (e.g., de la Fuente et al., 2021; Joyce, 2017). The study of Kordikova (2002) even varies strongly from Gaffney’s (1990) interpretation, by also invoking the presence of postparietals, temporals, and postfrontals. None of these studies justify these identifications in detail, but also do not provide critical comments on earlier identifications. However, even the near perfect 3D preservation of the *Proganochelys* specimen SMNS 16,980 does not allow for a clear verification of the presence of the supratemporal (pers. obs. SWE), and the asymmetric preservation in *P. talampayensis* raises at least some doubt for this species as well. For both species in question, alternative interpretations could potentially be supported, especially as the respective bone areas are slightly raised so that an interpretation of an osteoderm identity cannot be ruled out with certainty. The original proposition of a supratemporal for *P. quenstedtii* was likely influenced by outgroup comparisons of the time (e.g., Gaffney, 1990). Since then, several early, non-testudinatan stem turtles have been identified/proposed from the Triassic. These can potentially shed light on the evolution of the supratemporal. For example, Schoch and Sues (2018) explicitly rule out the possibility that the probable stem-turtle *Pappochelys rosinae* had a supratemporal, due to the geometry and sutural contacts between the squamosal and parietal. Similarly, the well-preserved *Eurhynchochelys sinensis* has no supratemporal (Li et al., 2018). Its presence has been tentatively inferred for *Odontochelys semitestacea* (Li et al., 2008), which is, however, difficult to interpret with regard to its cranial sutures so that we regard this for now as an uncertain interpretation. In the few phylogenies that include all currently known proposed non-testudinatan stem turtles (e.g., Li et al., 2018), *P. rosinae*, *E. sinensis*, and *O. semitestacea* are in a more stemward position of the turtle lineage than *Proganochelys*, suggesting that the supratemporal may have been lost already by the time Testudinata evolved. Thus, *Eunotosaurus africanus* from the Permian of South Africa is the only putative stem turtle with a certain supratemporal (Bever et al., 2015), although it should be noted that recent taxonomically comprehensive phylogenies do not actually support this taxon to be a stem turtle (Simões et al., 2022). Our CT scans of SMF 09-F2 show no internal suture in the parietal–squamosal area that would unambiguously attest the presence of a supratemporal, but given internal sutures are generally obliterated in the material, this cannot be seen as strong evidence for the absence of the element. However, we advocate that the specimen SMNS 16980 should be re-examined to specifically address this question, possibly with X-ray techniques, such as neutron scanning.

Medial to the expected supratemporal position, the parietals of the Frick skull show prominent dorsal protuberances at the posterior skull margin (Figs. 4, 5). Similar protuberances can also be seen in the *Proganochelys quenstedtii* material described previously (Gaffney, 1990: scale 8 in SMNS 15759 and SMNS 16980; figs. 17, 35, 36, 38, 39). The protuberances are slightly asymmetrical in the new fossil skull (Figs. 4, 5). On the right side of SMF 09-F2, there is a single, mount-like protuberance (Figs. 4, 5). The respective left side structure consists of two smaller and less regular protuberances that are separated by a transverse groove (Fig. 4B). Our CT data show that all three protuberances (which would correspond to scales 8 and 9 in SMNS 15,759 and SMNS 16,980: Gaffney, 1990; p. 29) are internally traversed by a dense network of neurovascular canals, which we partially segmented (Fig. 5). These canals seem to radiate from the centrum of the protuberance and furcate toward the outer bone surface, where they exit through many small foramina on the skull roof. There are also a few small entry foramina on the ventral surface of the skull roof, which are positioned in cavity-like fossae (Fig. 4F). Similarly, intense networks of neurovascular canals are usually only known from the beak region of turtle skulls, associated with the rhamphotheca. We, therefore, hypothesize that the protuberances are essentially horn cores, and that the observed canal system was to innervate a keratinous structure that covered the parietal protuberances.

The supraoccipital of SMF 09-F2 only has a very weakly developed median keel on the posterodorsally exposed occipital surface of the bone (Fig. 5A, C), contrasting a stronger keel in SMNS 16980 (Gaffney, 1990). SMF 09-F2 has a very prominent basis tuberculi basalis, which forms a dorsally tall projection on the floor of the braincase (Figs. 5C, 6). There is a low but distinct median ridge that originates at the basis tuberculi basalis and which extends for a short distance anteriorly along the dorsal surface of the basisphenoid. In previously described *Proganochelys quenstedtii* material (SMNS 16980), the basis tuberculi basalis is unremarkable and not associated with a ridge (Gaffney, 1990).

**Fig. 7:**
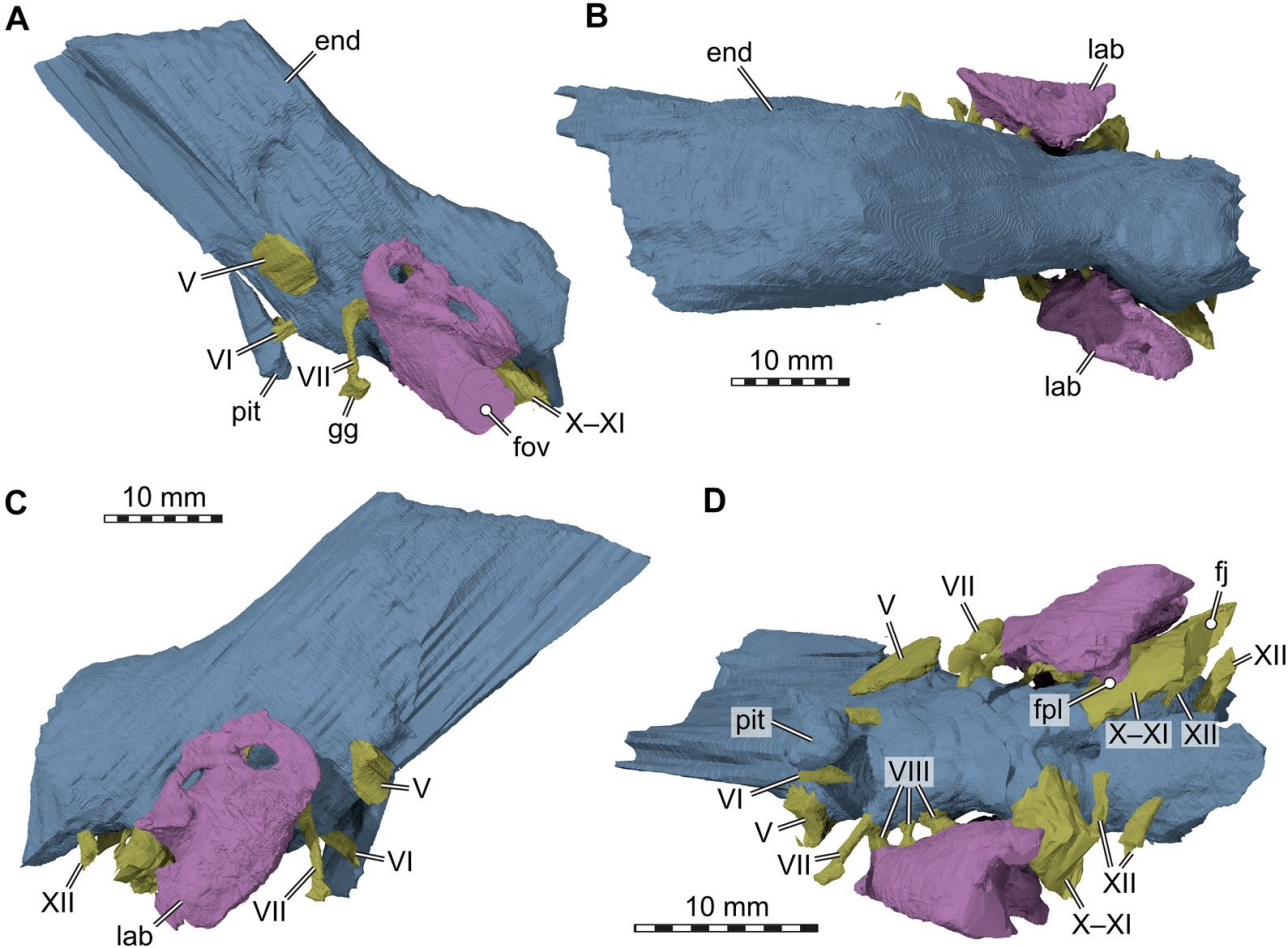
3D renderings of the braincase endocast, cranial nerve endocasts, and endosseous labyrinth model of *Proganochelys quenstedtii* (SMF 09-F2). **A,** Left lateral view; **B,** Dorsal view; **C,** Right lateral view; **D,** Ventral view. Abbreviations: end, cranial endocast; fj, foramen jugulare; fov, fenestra ovalis; fpl, fenestra perilymphatica (interface between endosseous labyrinth and canal for the CN X–XI); gg, geniculate ganglion of the facial nerve (CN VII); lab, endosseous labyrinth; pit, endocast of the pituitary fossa; V, trigeminal nerve (CN V); VI, abducens nerve (CN VI); VII, facial nerve (CN VII); VIII, acoustic nerve (CN VIII); X–XI, vagus nerve (CN X) and accessory nerve (CN XI); XII, hypoglossal nerve (CN XII)

**Fig. 8:**
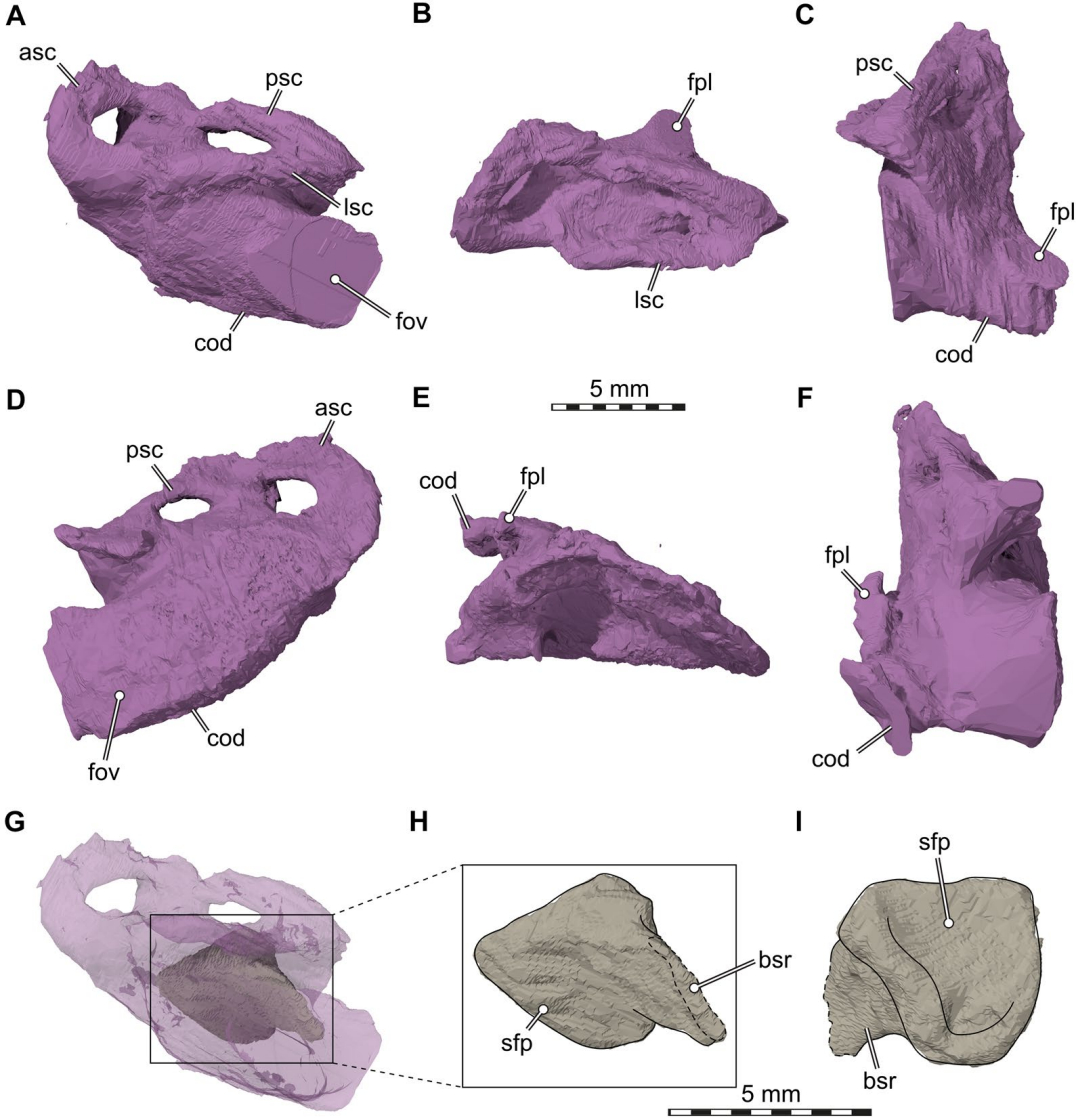
3D renderings of the endosseous labyrinths and stapedial fragment of *Proganochelys quenstedtii* (SMF 09-F2). **A,** Left labyrinth in lateral view; **B,** In dorsal view; **C,** In posterior view; **D,** Right labyrinth in lateral view, **E,** In dorsal view; **F,** In posterior view; **G**, Left labyrinth in lateral view rendered transparent to show preservational position of the stapedial fragment; **H**, Close-up of stapedial fragment in lateral view. **I**, In medial view. Note that the right lateral semicircular canal is broken. Abbreviations: asc, anterior semicircular canal; bsr, base of stapedial rod; cod, cochlear duct; fov, fenestra ovalis; fpl, fenestra perilymphatica; lsc, lateral semicircular canal; psc, posterior semicircular canal; sfp, stapedial footplate

Our CT data reveal several interesting features regarding the inner ear and anatomically related structures. The inner ear cavity of SMF 09-F2 is extremely well-ossified (Fig. 6). As is typical for early stem turtles, the fenestra ovalis is completely framed by bone laterally (Evers & Benson, 2019; Fig. 4C, D), and the fenestra is set in a small fossa we herein call the antrum of the fenestra ovalis (Fig. 4C–F). This morphology contrasts with more crownwardly positioned turtles, in which the fenestra ovalis is embedded within the space of the cavum acustico-jugulare. Besides being laterally well-encased, the inner ear cavity of SMF 09-F2 is also medially completely separated from the endocranial cavity by a vertical wall of bone, so that the hiatus acusticus is completely closed (Fig. 6). The foramina aquaducti vestibuli are small openings in this wall, and formed by the supraoccipital (Fig. 6). In previously described *Proganochelys quenstedtii* material (SMNS 16,980), the hiatus acusticus remains unossified instead (Gaffney, 1990). SMF 09-F2 shows clear evidence for the presence of perilymphatic fenestrae (Fig. 6), which connect the inner ear cavity with the recessus scalae tympani in anatomically modern turtles (e.g., Foth et al., 2019; Gaffney, 1979). A previous study describes the fenestra perilymphatica for *Proganochelys quenstedtii* as if it is present (Gaffney, 1990: p. 80) but then contradicts this previous description and argues in the same paper that it is not present (Gaffney, 1990: p. 87). It is the latter interpretation that since has been cited as the anatomical consensus (e.g., Foth et al., 2019; Sobral et al., 2016), but our observations (Figs. 7, 8) clearly demonstrate the presence of these openings, which can also be seen in the endocasts of the endosseous labyrinths (Fig. 8). In SMF 09-F2, the fenestra perilymphatica opens in the posterior otic wall formed by the processus interfenestralis of the opisthotic (Fig. 6), and thus in its expected topological position (Gaffney, 1979). It is clearly separate from the foramen jugulare anterius (Fig. 6), which corresponds to the remainder of the embryologic metotic fissure (Rieppel, 1985) and through which the vagus (CN X) and accessory (CN XI) nerves pass in turtles (Evers et al., 2019; Ogushi, 1913; Shiino, 1912; Soliman, 1964). In anatomically modern turtles, the foramen jugulare anterius is the medial opening of the recessus scalae tympani into the braincase (Gaffney, 1972, 1976, 1979), but the latter term is only applied once a lateral expansion of the exoccipital posteriorly encloses the space posterior to the fenestra interfenestralis (Anquetin et al., 2009; Gaffney, 1990). In SMF 09-F2, the recessus scalae tympani remains unossified (= foramen jugulare intermedius condition of Sterli & Joyce, 2007), which is in accordance with the morphology of other specimens of *Proganochelys quenstedtii* (Gaffney, 1990). As a consequence of the incompletely ossified recessus scalae tympani, the hypoglossal nerve (CN XII) foramina of SMF 09-F2 are situated on the lateral surface of the exoccipital facing laterally (rather than on the posterior surface of the laterally expanded exoccipital), slightly posteriorly to the position of the foramen jugulare anterius (Figs. 6, 7).

Although we specifically looked for a canal for the glossopharyngeal nerve (CN IX) in SMF 09-F2, we could not identify this structure, which apomorphically extends through the base of the processus interfenestralis in turtles (Rieppel, 1985), including in previously described *Proganochelys quenstedtii* material (Gaffney, 1990). We interpret this as a preservational artefact, rather than evidence for the absence of a bony glossopharyngeal canal. The canals for the facial (CN VII) and acoustic (CN VIII) nerves are clearly discernible in our CT data of SMF 09-F2, and show that three separate acoustic nerve rami were present in separately ossified canals (Fig. 6). The anteriormost of these is associated with the facial nerve canal, and only branches away from it within the prootic, whereas the posterior two acoustic nerve foramina are entirely separate, anteroposteriorly aligned openings. This is best appreciated in models of the endocast and nerves (Fig. 7). The acoustic and facial nerve foramina are not positioned in a fossa acoustico-facialis, which is a depression in the internal braincase wall of modern turtles (Gaffney, 1979). The facial nerve canal of SMF 09-F2 extends posterolaterally through the prootic in a laterally bowed trajectory (Fig. 7), and opens in the lateral surface of the braincase (Fig. 4). Specifically, this external facial nerve foramen is located slightly above the approximated position of the prootic–basisphenoid contact, and lies in the anterodorsal rim of a deep lateral fossa that stretches along the lateral braincase surface and which we call prootic fossa herein (Fig. 4C–F). This rim forms an overhanging ridge over the foramen, which we labelled as an orbitosphenoidal crest (Fig. 4D) in allusion to similar morphologies of some archosaurs. As a consequence, the facial nerve foramen can only be seen in posteroventral, not in strict lateral view of the braincase (Fig. 4). The lateral prootic fossa is difficult to appreciate in *Proganochelys* material when the quadrate is articulated (i.e., SMNS 16980; Gaffney, 1990). Gaffney describes two facial nerve foramina for SMNS 16980, one for the hyomandibular branch and one for the palatine branch of this nerve (Gaffney, 1990: fig. 27). This would indicate that the geniculate ganglion from which both rami diverge is positioned within the prootic (Rollot et al., 2021). Our specimen contradicts this, as the facial nerve canal clearly remains undivided before exiting the prootic posterolaterally. This morphology implies an extracranial ganglion position for *Proganochelys* along the lateral braincase wall and in the open cranioquadrate space, which is close to the canalis cavernosus in later stages of turtle evolution. Re-examination of the *Proganochelys* specimen that was used to inform on the description of the facial nerve (SMNS 16980: Gaffney, 1990) shows that the area of the facial nerve exit is elongated to a stretched fossa that is delimited by a sharp ridge, as is also the case in the new material SMF 09-F2, giving the expression of several aligned foramina. Externally, both specimens thus agree in their morphology surrounding the facial nerve exits. We propose that separate exists for the facial nerve rami in *Proganochelys quenstedtii* (Gaffney, 1990) are a misinterpretation based on the elongated fossa surrounding a singular foramen. It is likely that the facial nerve formed the geniculate ganglion right in this fossa, and that the anteroposterior elongated nature of the fossa is an osteological correlate for the anteriorly directed palatine branch and the posteriorly directed hyomandibular branch, which would have diverted from the geniculate ganglion. Similar ridges or grooves are often found associated with the hyomandibular branch of the facial nerve in extant turtles, whereas the anterior palatine branch is usually completely encased in bone (Rollot et al., 2021). Our interpretation agrees with previous observations (Lautenschlager et al., 2018) of the facial nerve branching outside of the braincase on the specimen MB 1910.45.2, but that study did not mention that this observation contradicts the primary description of the facial nerve anatomy of *Proganochelys quenstedtii* (Gaffney, 1990).

The abducens (CN VI) nerve foramina of SMF 09-F2 are also clearly identifiable canals, which traverse the basisphenoid anteroposteriorly at the dorsum sellae and exit the bone anteriorly halfway along the dorsoventrally deep pituitary fossa (Figs. 4, 6, 7). The clinoid processes of SMF 09-F2 are robust, dorsally projecting structures (Figs. 4, 5) in accordance with the morphology of other specimens (Gaffney, 1990). The left clinoid process is nearly completely preserved, and is expanded toward its dorsal tip, thereby nearly closing the primary trigeminal (CN V) foramen or fenestra prootica by a contact of the clinoid process and the pila antotica (Bhullar & Bever, 2009; Evers et al., 2019; Gaffney, 1990; Figs. 4, 5). The preserved morphology suggests that the foramen was fully ossified, as in other well-preserved *Proganochelys quenstedtii* specimens (Bhullar & Bever, 2009; Gaffney, 1990). It is noteworthy that the fenestra prootica is not homologous with the trigeminal foramen of anatomically modern turtles, which is secondarily formed as the braincase wall becomes expanded in turtles (Evers et al., 2019).

The cerebral branch of the internal carotid artery enters the basicranium through the basisphenoid in *Proganochelys quenstedtii* (Gaffney, 1990; Müller et al., 2011), and the turtle cerebral blood circulation only becomes internalized in later evolutionary stages (Rabi et al., 2013; Rollot et al., 2021; Sterli et al., 2010). Besides the two expected foramina posterius canalis carotici basisphenoidalis (following the nomenclature of Rollot et al., 2021) for the right and left arteries, SMF 09-F2 has a third, centrally placed foramen (Fig. 4F), which we interpret as abnormal individual variation of the specimen. Whereas the two ‘regular’ canalis caroticus basisphenoidalis are symmetrical and converge anterodorsally within the basisphenoid, the central canal is shorter and connects internally with the left cerebral canal. In SMF 09-F2, the anterodorsal portion of both main cerebral canals join just before exiting into the sella turcica, thus forming a single anterior exiting foramen. This slightly contradicts the previous report (Gaffney, 1990) that the foramina would be very close together but separated. Previous carotid segmentation models of *Proganochelys quenstedtii* (Lautenschlager et al., 2018) are not conclusive in this regard as only one arterial canal from one side was fully segmented and as the carotid courses are not further discussed in that work. However, the exiting position of the fully segmented canal in a median (i.e., skull midline; Lautenschlager et al., 2018: fig. 1H) position provides tentative evidence that the canals indeed may merge to form a single, central exit foramen.

The partial endocast that we could segment from SMF 09-F2 includes the hind- and midbrain regions, but more anterior areas around the olfactory region are not preserved (Fig. 7), as the frontals are disarticulated from the parietals. However, the frontals are preserved separately, and indicate that weak crista cranii are present, marking the course of the olfactory tract (Fig. 3H, I). The olfactory lobes themselves do not leave impressions in the bone (Fig. 7B). This is consistent with previous brain endocast descriptions for *Proganochelys quenstedtii* (Lautenschlager et al., 2018). As these authors have also noted, the we find moderate cranial flexures in the braincase endocast, and no clear demarcations of any brain regions (Fig. 7), suggesting that *Proganochelys quenstedtii* had a similarly poor brain tissue to endocranial cavity correspondence as has been documented for extant turtles (Evers et al., 2019; Werneburg et al., 2021; Wyneken, 2001).

The endocasts of the endosseous labyrinth of SMF 09-F2 (Fig. 8) are also remarkably similar to previously published models for other *Proganochelys quenstedtii* specimens (Lautenschlager et al., 2018). This is important to note, because a prominent feature of the labyrinth—a notable posterior displacement of the fenestra ovalis with regard to the semicircular canal system (Fig. 8A, D)—is unusual for turtles and may have been interpreted as a likely preservational artefact. Our symmetrically preserved labyrinth models and those provided previously (Lautenschlager et al., 2018) demonstrate that this posterior displacement is an original feature. In most extant and extinct turtles, the fenestra ovalis can clearly be traced in endosseous labyrinths, and it is usually positioned centrally in the labyrinth, and ventral to the level of the semicircular canal system (Evers et al., 2019; Lautenschlager et al., 2018; Paulina-Carabajal et al., 2013). In *Proganochelys quenstedtii*, including SMF 09-F2, the centroid of the fenestra ovalis is positioned approximately at the level of junction of posterior and lateral semicircular canal (Lautenschlager et al., 2018; Fig. 8A, D). Similarly, the fenestra perilymphatica is positioned posterior to the semicircular canals, and not ventrally underneath it (Fig. 8B, C, E, F). This may be of functional importance, as the position of the fenestra perilymphatica usually is a good indicator of the extent of the cochlear duct, a structure that is usually short in turtles (Evers et al., 2019; Walsh et al., 2009). The posterior displacement of the fenestra perilymphatica in *Proganochelys* implies that the cochlear duct did not extend straight down, but was posteroventrally recurved from the labyrinth centroid, extending its length. Cochlear length is associated with hearing frequency sensitivity in extant reptiles and birds (Manley, 1972; Walsh et al., 2009), and long and posteriorly recurved cochlear ducts in extant birds are associated with nocturnality (Choiniere et al., 2021). We note herein, that we are not proposing nocturnal habits for *Proganochelys*, because such claims should be evaluated in a comparative statistical framework, as recently done for theropod dinosaurs (Choiniere et al., 2021). However, a possibly expanded cochlear length in *Proganochelys* with regard to other turtles offers potentially new research lines into its sensory ecology, and at least implies unappreciated sensory disparity in the cochleae of stem turtles.

The semicircular canal system of SMF 09-F2 agrees with previous description (Lautenschlager et al., 2018): the labyrinth has a low aspect ratio (i.e., dorsoventrally low but anteroposteriorly elongate), is mediolaterally narrow, and the semicircular canals are short and well-rounded (Fig. 8). Posterior and lateral semicircular canals form a secondary common crus in the posterior part of the labyrinth (Fig. 8C, F) as in all known turtles (Evers et al., 2019), and the canals a moderately robust in terms of their internal diameter. In SMF 09-F2, the right lateral semicircular canal is internally broken and could not be reconstructed in full (Fig. 8C).

Within the left inner ear cavity, SMF 09-F2 preserves the medial part of a stapes, which must have been pushed into the inner ear and preserved within (Fig. 8G). The preserved part is formed as an expanded footplate (Fig. 8H–I), and preserved a short base of a stapedial rod, which appears crushed and flattened (Fig. 8H). This contradicts previous speculations that the stapedial footplate of *Proganochelys quenstedtii* was not expanded (Gaffney, 1990), based on the thick nature of the stapedial rod in the Berlin skull.

### Axial skeleton

#### Shell

**Fig. 9:**
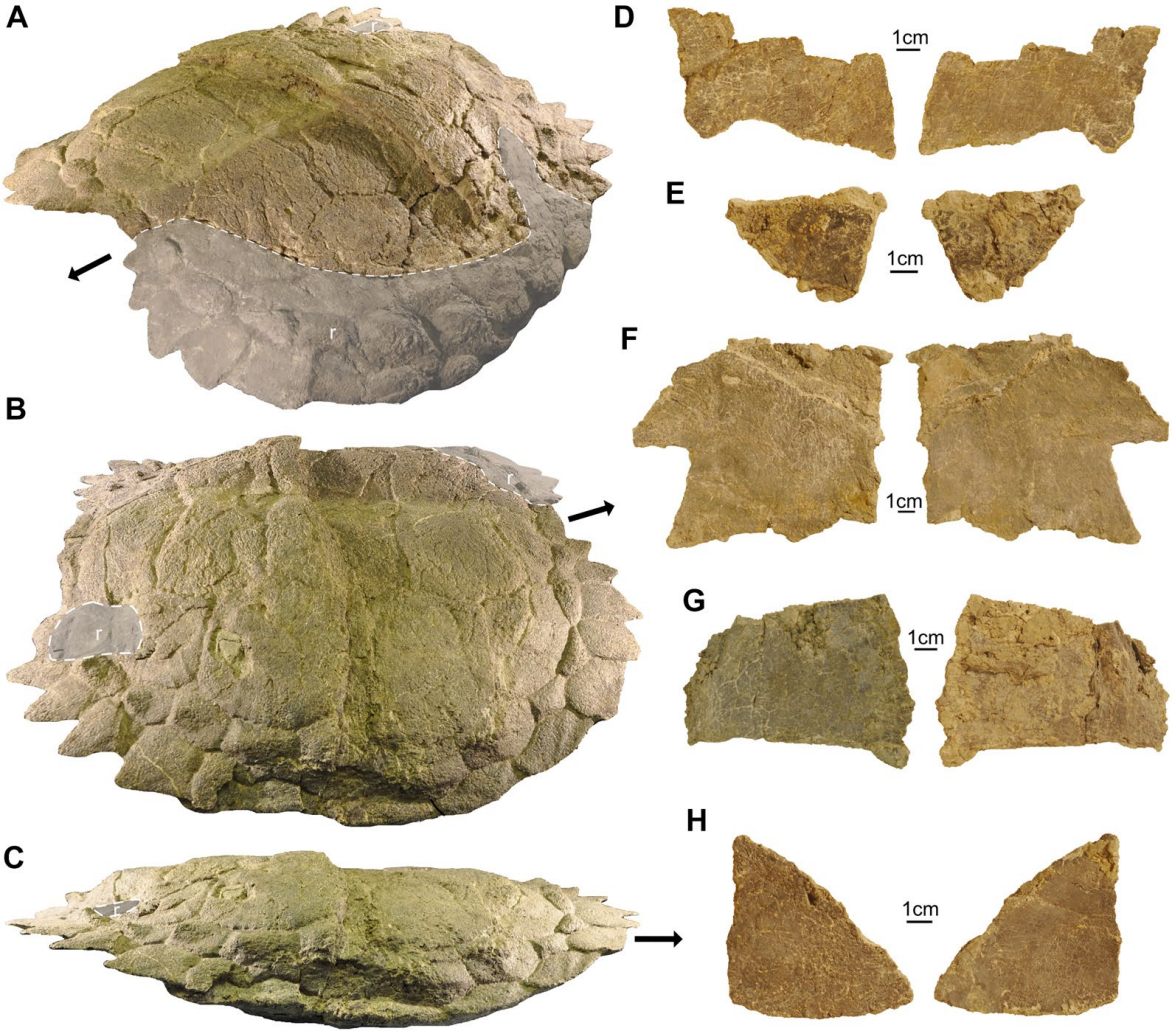
Shell remains of *Proganochelys quenstedtii* (SMF 09-F2). **A–C,** Carapace (**A** angled anterodorsolateral, **B,** right side in dorsolateral, **C,** right side in lateral view). Reconstructed parts of the carapace are delimited by grey-shaded area set off by a stippled white line and marked with a white r. The anterior aspect of the carapace is marked in each view by a black arrow. **D–H,** Various parts of the plastron. Due to the fragmented nature of these bones, their position in the plastron is ambiguous

The carapace is prepared and reconstructed now displaying the dorsal side (Fig. 9; Additional file 3: Fig. S3). The right side of the carapace is preserved completely, while parts are missing on the left side. A break of approximately 18–20 mm splits the complete carapace from the left to the right side. In addition, the carapace displays a prominent transverse crack that leads to an overlap of the posterior carapace portion onto the anterior portion by approximately 30 mm on the right side. The midline straight carapace length (SCL) as preserved, i.e., without this overlap of the carapace parts, is 51.9 cm. The preserved straight carapace width (SCW) is 55 cm and the reconstructed SCW (by mirroring the more complete right side) is 61 cm. The carapace is well-ossified and sutures between carapacial elements are not or only very weakly identifiable (e.g., between few peripheral elements). Only in visceral view, some (neuro-)vascular grooves that seem to be linked with the original presence of sutures between some costals of the mid-carapacial region had been visible before the carapace was mounted for exhibition (Additional file 4: Fig. S4); altogether nine costals had been counted. The overall morphology of the carapace, especially the shield sulci, resemble that described for the German specimens of *Proganochelys quenstedtii* (Gaffney, 1990). There seem to be differences in the proportion of the peripherals and supramarginals, which could, however, be due to taphonomic distortion, and the proportional differences seem to still lie within the intraspecific range for this species. Mainly the size of the spike-like projections of the posterior peripherals seems to be variable (Gaffney, 1990; Fig. 9A–C), which could, however, lead to different counts of the marginals. Shape variation in the marginals is also a common feature and well-documented, for example, in specimens of *Proterochersis* (Szczygielski et al., 2018).

In the costals, the proximal rib and the rib head are well set apart from the costal. The nuchal region of SMF 09-F2 is only incompletely preserved and sutures are not discernible here. The visible nuchal embayment is moderate with comparable dimensions to the other *Proganochelys* specimens (Gaffney, 1990; Fig. 9A). The embayment of the pygal region is of similar width but maybe not as deep as in other specimens from Trossingen.

On the right side of the carapace, 12 supramarginals are visible (the 12^th^ being reconstructed) and 15 marginals with the 10^th^ broken and overlapping the 9^th^ approximately 3 cm (Fig. 9A–C; if a potential 16^th^ marginal would be present, it would only rudimentarily be developed and lacking a distinct spike). SMF 09-F2 thus appears to differ in the number of marginals compared to SMNS 16980 and MB 1910.45.2 which, according to Gaffney (1990) have 16 or 17 marginals, respectively. One cervical shield, four vertebrals, one supracaudal and four pleurals are present, as was described for *Proganochelys quenstedtii* (Gaffney, 1990).

In ventral view, there are several mediolaterally extending shallow grooves present between the second and third, third and fourth, and fourth and fifth thoracic rib (Additional file 4: Fig. S4). These grooves are similar to the ones described in other *Proganochelys* specimens, either interpreted as being grooves for nerves or vessels, that are at similar position as intercostal sutures (Gaffney, 1990: p. 123–124) or representing remains of true intercostal sutures, as was originally proposed (Jaekel, 1916), with the sutures being in the process of becoming completely fused and thus more and more obliterated.

The bridge region is completely missing on the left side of the shell and not very well-preserved on the right side. It seems to extend from the fifth to the eleventh marginal, which is on the same level as the 1^st^ to 7^th^ costal. Several fragments of the plastron (Fig. 9D–H) have been preserved that are not articulated and difficult to assign to specific plates. A striking feature is the extreme thinness (3–5.5 mm) of these plastral remains, which is comparable with other *Proganochelys* specimens (e.g., Gaffney, 1990) and also other Triassic stem turtles, such as *Waluchelys, Palaeochersis*, and *Proterochersis* (e.g.Sterli et al., 2007, 2021; Szczygielski & Sulej, 2016, 2019; Szczygielski et al., 2018).

#### Vertebral column

**Fig. 10:**
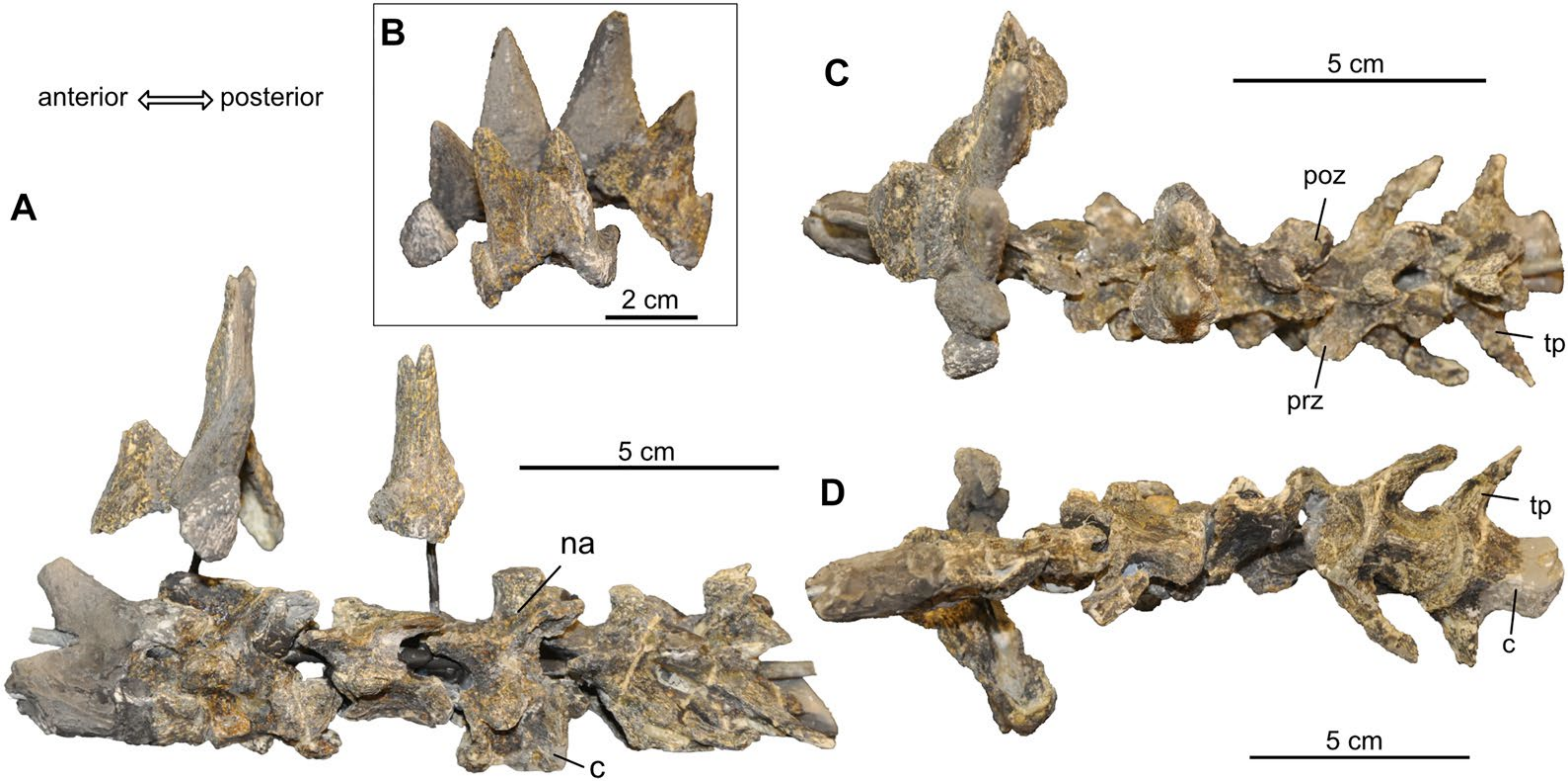
Neck vertebral column of *Proganochelys quenstedtii* (SMF 09-F2). **A,** Cervical vertebrae and osteoderms in left lateral view. **B,** Osteoderms as they are mounted over the cervical vertebrae in posterior view. **C,** Cervical vertebrae and osteoderms in dorsal view. **D,** Cervical vertebrae and osteoderms in ventral view. Abbreviations: c, centrum; na, neural arch; poz, postzygapophysis; prz, prezygapophysis; tp, transverse process

All cervical vertebrae, found partially disarticulated, are preserved, although most are heavily deformed (Fig. 10) and thus provide little information on the original vertebral shape. The atlas elements are highly eroded and have been reconstructed for mounting, but they are still articulated with the axis, which is easily identified due to its elongated neural spine. Cervicals 3–7 are identified due to successively more elongated cervical ribs toward posterior (compare to Gaffney, 1990: fig. 108). The cervicals are accompanied by two sets of osteoderms consisting of dorsally tapering spikes with rounded or oval bases; the first is partially reconstructed, the second is completely preserved (Fig. 10A–C). There is no indication that the spikes consist of smaller sutured elements, nor were more than two sets of neck spikes found (the latter thus further supporting the interpretation of Werneburg et al., 2015, contra to Gaffney’s, 1990 life reconstruction carrying four sets of spikes).

Ten thoracic vertebrae are preserved with the carapace. The 9^th^ and 10^th^ are the least well-preserved together with the 6^th^, where the crack splits the specimen in an anterior and posterior half. Prezygapophyses and articular facets were generally not well visible during the preparation process. The last three pairs of thoracic ribs are only very poorly preserved. The rib heads are set off from the costals and are very broad with a strong articulation to the vertebrae. The scapular pit is visible on the left side of the shell anterior to the first thoracic rib and posterior to a small ridge on the carapace that reached the epiplastral process (Additional file 4: Fig. S4). Two sacral vertebrae and ribs (described below) are still in articulation with the pelvic girdle.

**Fig. 11:**
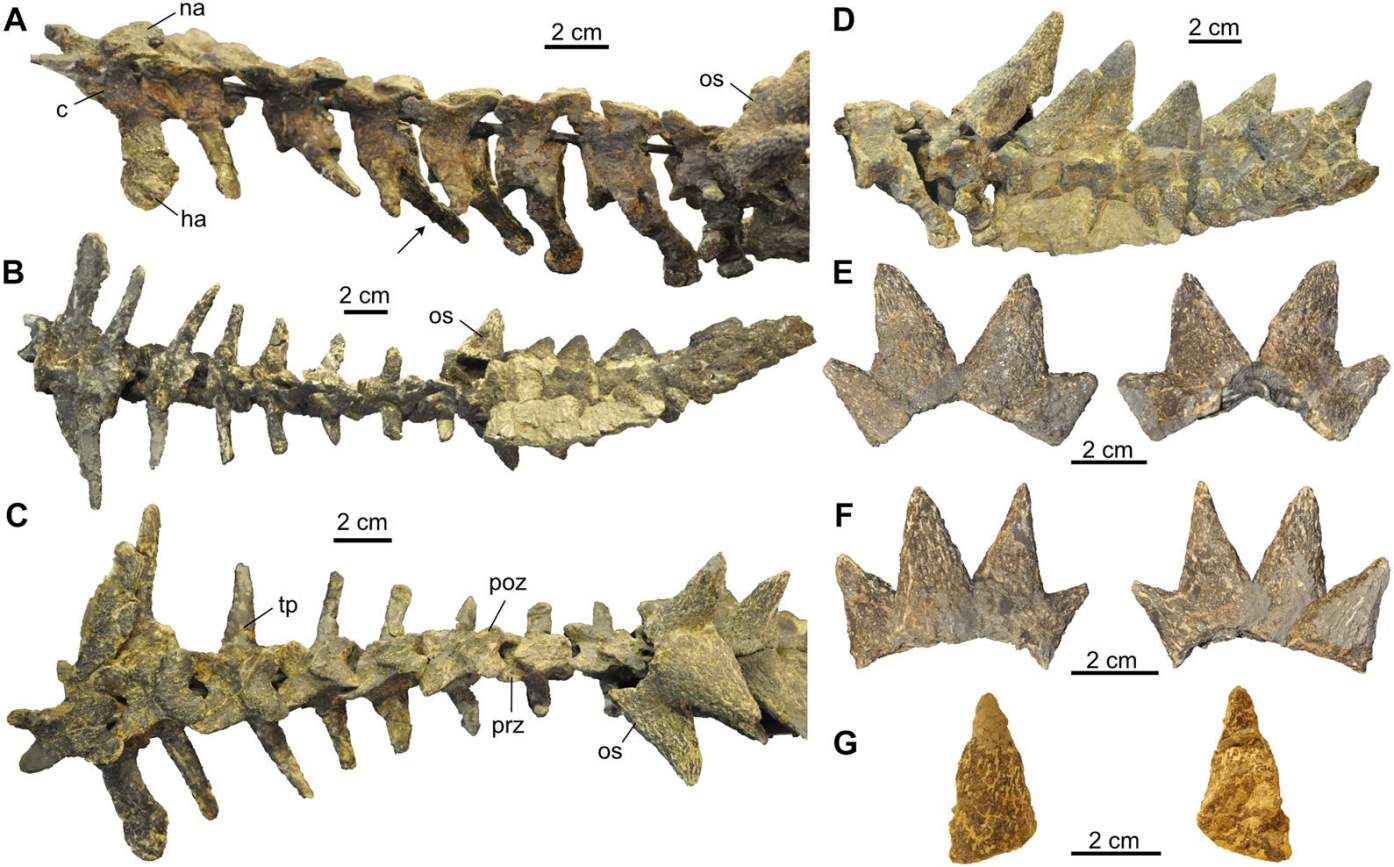
Tail region of *Proganochelys quenstedtii* (SMF 09-F2). **A,** Preserved caudal vertebrae in left lateral view. The black arrow points at the hemal arch of the 4^th^ preserved caudal vertebra from anterior. **B,** Preserved tail in ventral view. **C,** Preserved caudal vertebrae and anterior osteoderms of the tail club in dorsal view. **D,** Tail club in left lateral view. **E, F,** Individual osteoderms consisting of four spikes each, in anterior and posterior views. The exact position of these elements anterior to the tail club (i.e., closer to the base of the tail) is ambiguous. **G,** Isolated tail spike in anterior and posterior view. Abbreviations: c, centrum; ha, hemal arch; na, neural arch; os, osteoderm; poz, postzygapophysis; prz, prezygapophysis; tp, transverse process

At least twelve caudals are associated with the specimen that are largely covered posteriorly by a tail club of which only the posterior-most tip is reconstructed (Fig. 11). The size of the transverse processes decreases from anterior to posterior and the neural arches are very low. From the 4^th^ caudal onward (Fig. 11A), chevron bones are preserved that are situated at the posteroventral margin of the centra. The tail of SMF 09-F2 shows four spiked osteoderms and two additional osteoderms, each with four spikes that likely were part of the tail as well (Fig. 11B–F). An additional isolated spike (Fig. 11G) could be recovered as well, but its position on the tail is unclear. The tail as preserved is 35.8 cm long. For comparison, one specimen from Trossingen (SMNS 17204) shows seven spiked osteoderms associated with the tail (Gaffney, 1990).

### Appendicular skeleton

#### Pelvic girdle

**Fig. 12:**
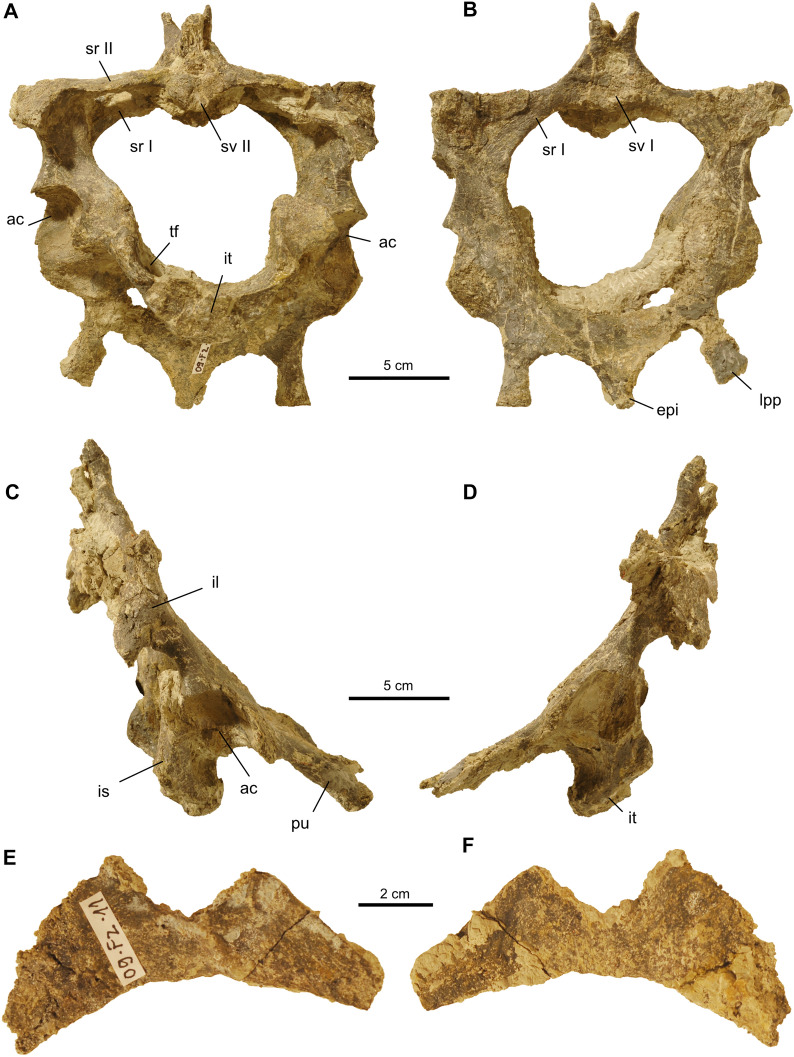
Pelvic girdle and sacrum of *Proganochelys quenstedtii* (SMF 09-F2). **A–D,** Pelvic girdle consisting of fused pubes, ilia and ischia (A posterior, B anterior, C right lateral, D left lateral view). Note that the hole in the left pubis does not represent the thyroid fenestra but is a break in the bone. **E, F,** Fragment of the postpelvic hypoischium. Abbreviations: ac, acetabulum; epi, epipubic process, il, ilium; is, ischium; it, ischial tubercle; lpp, lateral pubic process; pu, pubis; sr I, first sacral rib; sr II, second sacral rib; sv I, first sacral vertebral centrum; tf, thyroid fenestra

The pelvis is completely preserved and the elements are still articulated to each other as well as fused to two sacral ribs and vertebrae (Fig. 12A–D). Sutures are not discernible, supporting the hypothesis that the pelvis of adult *Proganochelys* was completely ossified (Gaffney, 1990:183). The girdle is anteroposteriorly flattened as a consequence of the preservation and the processes and articulation facets are eroded and poorly preserved. The centrum of the first sacral is slightly concave anteriorly and the posterior articulation of the second sacral vertebra appears platycoelous, but the bone is damaged in this region making identification of the shape difficult. Above the neural canal, the prezygapophyses of the first sacral are forming a V (with a steep angle of about 65°) with the articular facets facing medially and almost touching each other ventromedially. The angle between the prezygapophyses is thus just slightly less steep than in the first sacral of MB 1910.45.3 and what has been generally described for *Proganochelys quenstedtii* (Gaffney, 1990: p. 186). Of the neural arches and spinous processes of both sacrals only the bases are preserved in SMF 09-F2. The first sacral rib is more robust and longer proximodistally than the second rib, as is also the condition in the specimens from Germany (Gaffney, 1990: p. 183). Both ribs encompass a large space in dorsal view.

The ilium is massive with an anteroposteriorly dorsal expansion. The dimensions of the anterior ilial process are as figured previously (Gaffney, 1990). The posterior ilial process is incomplete in SMF 09-F2 but dimensions seem to be similar to the German specimens as well. The oval tubercles of the ilia that articulate with the carapace are poorly preserved but visible. The pubes, which in SMF 09-F2 are ventrally bent, form the anterior half of the pelvic girdle in ventral view. Both pubes meet along the midline, where they ventrally form a pointed epipubic process that extends slightly beyond the anterior margins of the lateral pubic processes. The latter extend anteroventrally right and left to the epipubic process, making the articulation with the plastron. The ischium forms the posterior half of the pelvis in ventral view. The only well visible feature in SMF 09-F2 is an oval and prominent ischial tubercle representing the ventral midline. The lateral ischial processes are slightly concave in SMF 09-F2 and not with a straight margin as in the specimens from Germany (Gaffney, 1990). However, the morphology of this region seems to be quite variable in *Proganochelys* (Gaffney, 1990: p. 195). The thyroid fenestrae are still obscured by sediment. Because of deformation during fossilization, the right fenestra is located more posteriorly than the left and not parallel to the sacrum, but vertical. The tripartite Y-shaped suture of the three pelvic elements that form the acetabulum is also not visible. The acetabulum is deeply concave with well-formed margins (Fig. 12C, D).

In addition, a fragmented two-pronged flat bone with a crescent shaped margin is interpreted here to be a hypoischium (Fig. 12E, F). The shape conforms to the unpaired hypoischium shape of SMNS 16980 (Gaffney, 1990: p. 208) rather than the paired hypoischia of other specimens (e.g., SMNS 15759 and 17203) from Germany.

#### Forelimb

**Fig. 13:**
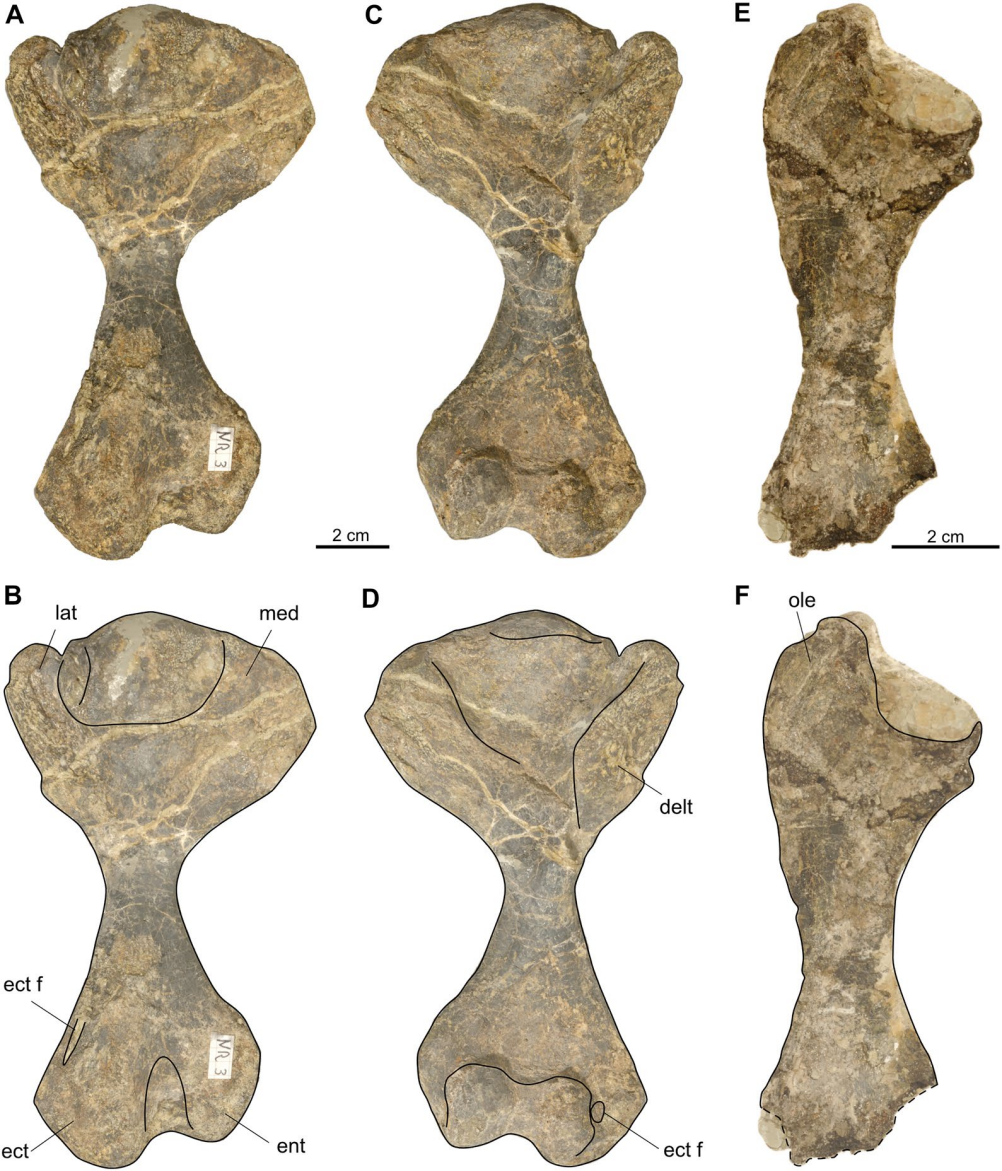
Forelimb elements of *Proganochelys quenstedtii* (SMF 09-F2). **A–D,** Left humerus and interpretative drawings superimposed (A, B dorsal, C, D ventral view). **E, F,** Left ulna and interpretative drawing superimposed in ventral view. Abbreviations: delt, deltopectoral crest; ect, ectepicondyle; ect f, ectepicondylar foramen; ent, entepicondyle; lat, lateral process; med, medial process; ole, olecranon

Of the forelimbs of SMF 09-F2, a left humerus and a left ulna (associated with few small plate-like osteoderms, not figured) were recovered (Fig. 13).

The left humerus (Fig. 13A–D) is completely preserved but dorsoventrally flattened except in the short shaft area. The humerus of *Proganochelys* resembles already the morphology of other turtles (Gaffney, 1990). It displays the typical proximal roughly hemispherical expansion and the somewhat narrower distal expansion connected by a straight shaft that, in anteroposterior view, is sigmoidally curved. Morphological details are as described in (Gaffney, 1990: fig. 149) including the presence of a sediment-filled slit-like ectepicondylar foramen visible in dorsal view.

The left ulna (Fig. 13E, F) is mostly complete but slightly damaged in the distal articular area. It is a bowed bone, with a prominent olecranon framing the semilunate articular facet for the entepicondyle of the humerus.

#### Hind limb

**Fig. 14:**
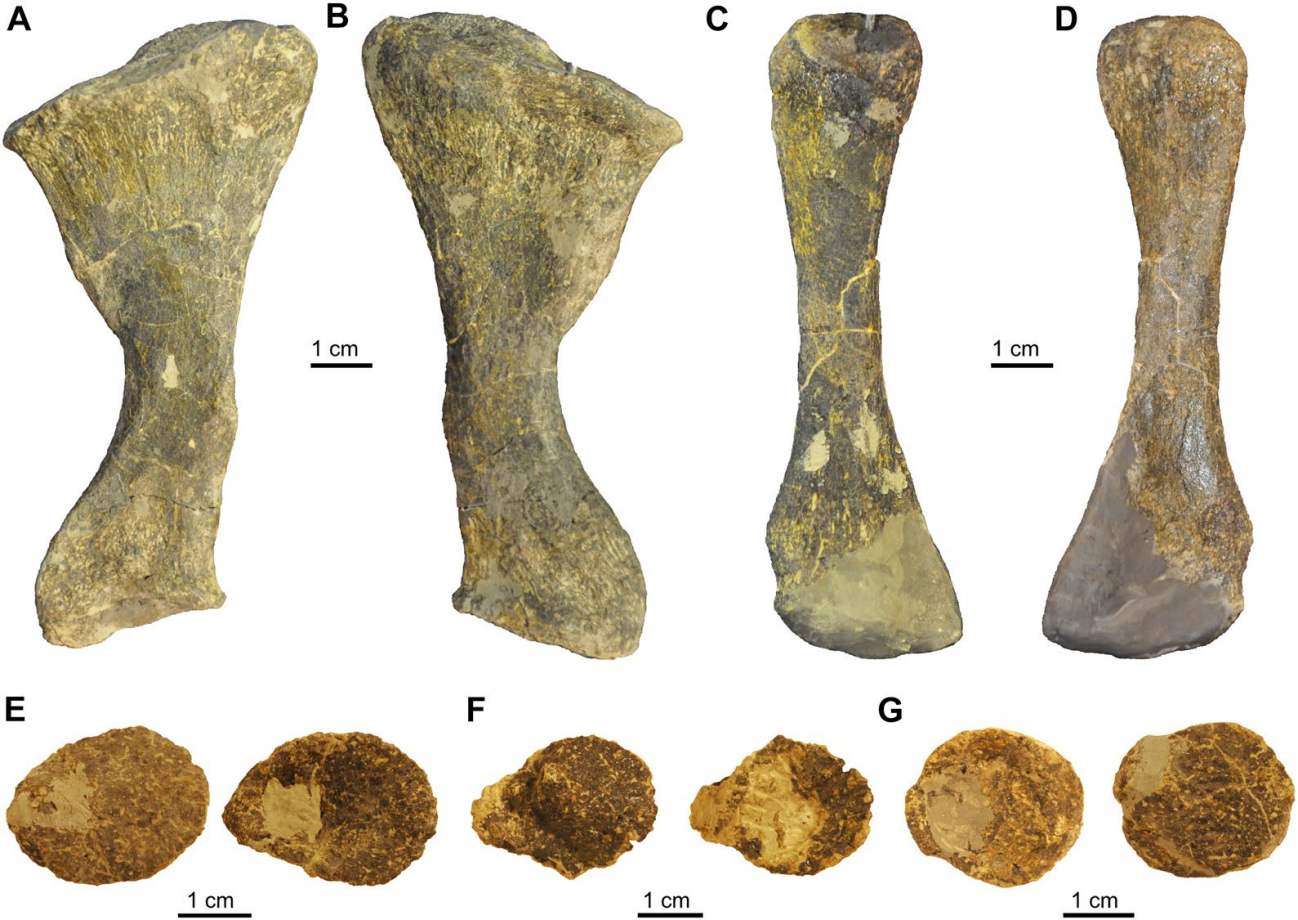
Hindlimb elements (zeugopodium) of *Proganochelys quenstedtii* (SMF 09-F2). **A, B,** Right tibia (A ventral, B dorsal view). **C, D,** Right fibula (C ventral, D dorsal view). **E–F,** Limb osteoderms with ovoid bases and off-centred peaks

**Fig. 15:**
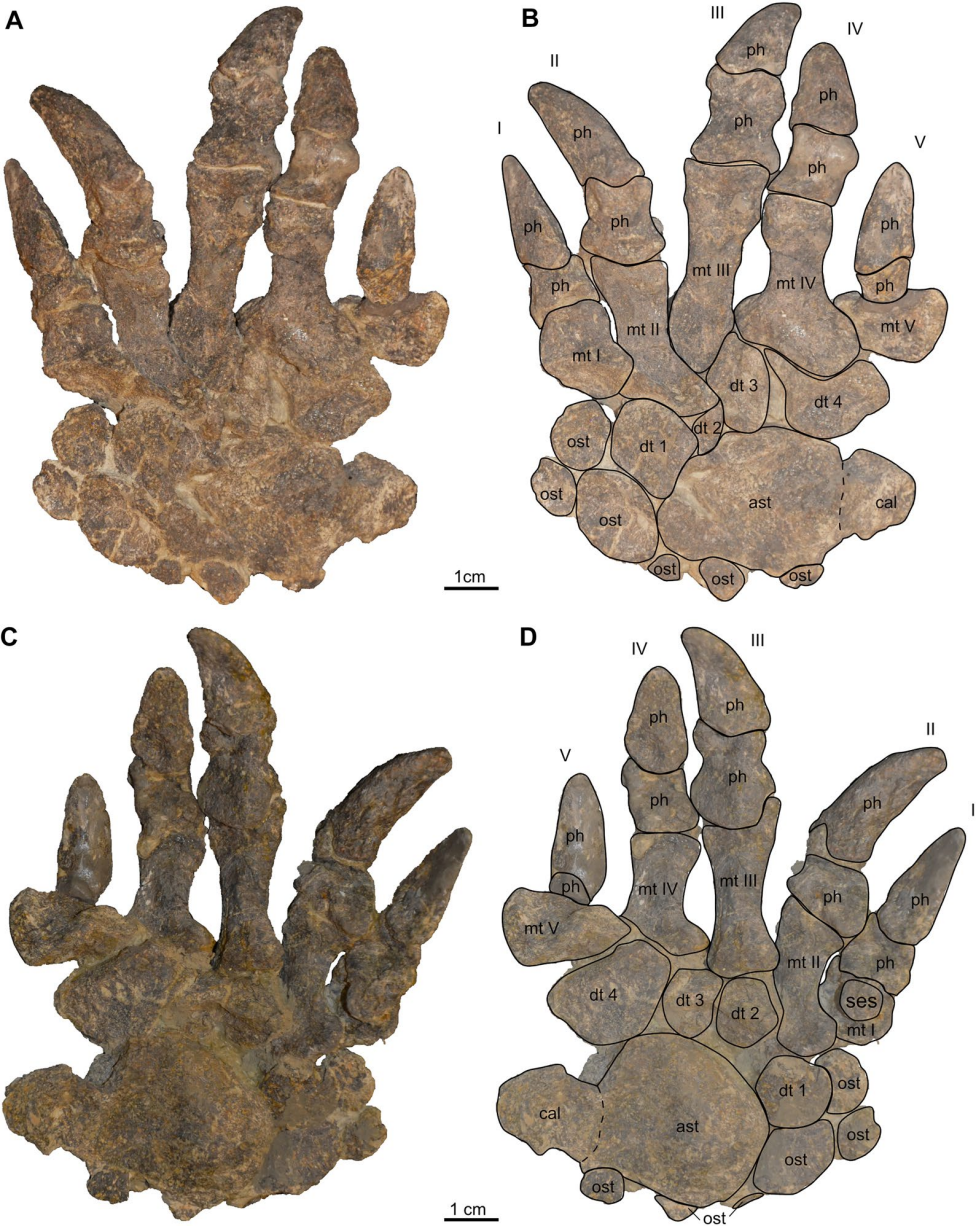
Hindlimb elements (left autopodium) of *Proganochelys quenstedtii* (SMF 09-F2). **A–D**, Articulated pes with interpretative sketches superimposed (A, B dorsal, C, D ventral/plantar view). Abbreviations: ast, astragalus; cal, calcaneum; dt 1–4, distal tarsal 1–4; mt I–V, metatarsal I–V; ost, osteoderm; ph, phalangeal bone; ses, sesamoid bone

From the hind limb, the right zeugopodium and autopodium were preserved on one slab and initially prepared on both sides of the slab, revealing also many limb osteoderms and sesamoids associated with the hind limb (Additional file 5: Fig. S5). The limb bones were later completely removed from the matrix block to be included in the museum exhibition (Figs. 14, 15).

In the zeugopodium, the tibia is massive and laterally curved, proximally wide, and has a narrow shaft (Fig. 14A, B). The tibia terminates in a narrow, tapering distal end. The fibula (Fig. 14C, D) is slenderer than the tibia and has a narrow proximal and a wide distal end, now partially reconstructed. Several osteoderms (described below) were found associated with the hindlimb (Fig. 14E–G; Additional file 5: Fig. S5).

The autopodium (Fig. 15A–D) is completely preserved and still in articulation. It consists of a massive astragalocalcaneum-complex followed by distal tarsals 1–4 and metatarsals I–V (note that metatarsal I appears ‘hooked’ in dorsal view but that is only based on the preservation and slight rotation of the bone in relation to digit 1). The metatarsal and the proximal phalanges are dumbbell shaped, with the former being much larger and with a distinct and elongated shaft. Each digit consists of short proximal phalanx and an ungual (terminal phalanx). The unguals are slightly curved and claw-like as they are broad and high proximally but narrow and tapering distally. As in the German specimens, the third digit appear longest in the pes.

**Fig. 16:**
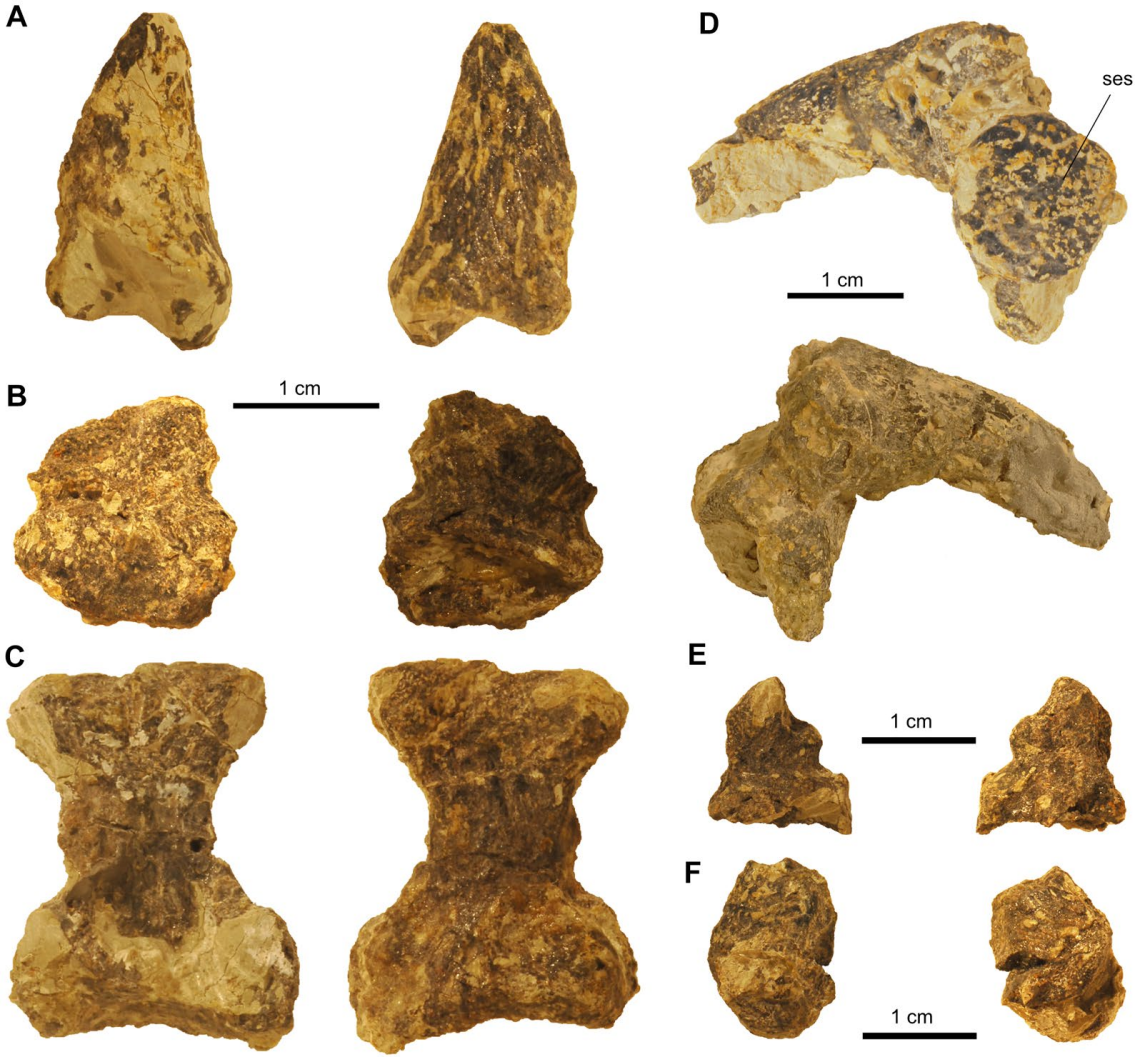
Isolated elements of the left pes of *Proganochelys quenstedtii* (SMF 09-F2). **A–C,** Isolated elements, potentially belonging to a single digit. **A,** Terminal phalange/ungual. **B,** Proximal phalangeal bone. **C,** Metatarsal bone. **D,** Articulated digit associated with sesamoid bone

In addition, disarticulated fragments pertaining to the left autopodium were recovered (Fig. 16). Given that these autopodial elements, associated with a few bones that likely represent osteoderms/sesamoids, were found on one block in close proximity to tail vertebrae and spiked osteoderms, they likely pertain to the other hindfoot. Of these bones, one metacarpal, two first/proximal phalanges, and two terminal phalanges could be identified. Of those elements, two phalanges are still in articulation, together with a round plate-like sesamoid (see below), forming one digit. The other digital elements were found isolated, so they potentially belong to separate digits.

#### Sesamoids and Osteoderms

Additional ossifications in the skin and connective tissue (i.e., tendons) of turtles are known from extinct and extant terrestrial tortoises and extinct non-testudinoid taxa (e.g., Joyce, 2017; Scheyer et al., 2015). For *Proganochelys quenstedtii,* Gaffney (1990) described sesamoid ossifications in the extensor tendons of the digits, amongst others, in the forefoot. We here identify similar sesamoids in SMF 09-F2 articulated with the first digit of the right pes (Fig. 15C, D) in the form of a circular flat bone on the ventral surface of metatarsal I, as well as a similar element associated with a phalange and ungual of the putative left pes. This bone also has a round shape and is attached to the phalanx, as is the case in the specimens from Germany (Gaffney, 1990). Six osteoderms are present attached to the articulated right pes, anterior and lateral to the astragalus and anterior to the first and second metatarsal (Fig. 15B, D). These bones vary in shape from roundish to elongate and all of them are flattened. Furthermore, there are six disarticulated limb osteoderms, four of them flat and roundish and two elongated with a conical tip, that were recovered from the block including the right hindlimb elements.

## Conclusions and discussion

Given the long history of scientific excavations at the clay pit Gruhalde in Frick since the mid-1970s (e.g., Sander, 1992), which yielded so far tens of well-preserved plateosaurs, as well as a selection of other usually more rare and smaller faunal elements, it might be surprising that no articulated turtle remains had been recovered from this important site. The recent recovery of plateosaurs and SMF 09-F2 from a ‘simple’ housing construction site on the other side of the valley at Frickberg, however, hints at the extent of the fossiliferous layers in the whole region and at the potential for retrieving fossils outside of the claypit. Similar to the other important Central European plateosaur-bearing bonebeds of Trossingen and Halberstadt, *Proganochelys quenstedtii* can be considered a rare faunal element in Frick.

The specimen SMF 09-F2 is inferred to have reached skeletal maturity based on humerus and carapace lengths that are in the upper range of other known individuals of *Proganochelys* (Gaffney, 1990). This inference is supported by the apparent degree of fusion of the shell and cranial bones, specifically in the posterior skull part. Individual bone sutures in the shell of the Swiss specimen are also all but obliterated and not traceable.

In the skull, we confirm a recurved/crescent-shape of the quadratojugal and show that the skull roof is well-vascularized along low tubercles, with numerous foramina opening up to the dorsal and visceral bone surface. The vascularisation is best explained by the presence of a keratinous cover, so that the tubercles can be interpreted as small horns that frame the posterior margin of the skull roof. In this regard, Gaffney (1990) discussed potential homology of the protuberances of the posterior skull roof at the region of scale 8 in *Proganochelys quenstedtii* with scale A in *Meiolania platyceps* skulls (see Gaffney, 1983b; Sterli, 2015).

In the inner ear region, we also show the presence of perilymphatic fenestrae, which connect the inner ear cavity with the space between the foramen jugulare anterius and the cranioquadrate space. As the latter ossifies more intensely during later stages of turtle evolution, the described space becomes the recessus scalae tympani (Rieppel, 1985). The presence of perilymphatic fenestrae contradict most recent interpretations of that region (Clack & Allin, 2004; Gaffney, 1990; Gaffney & Kitching, 1995; Sobral et al., 2016; Sterli & Joyce, 2007). Perilymphatic fenestrae of (crown) turtles are involved in the re-entrant fluid flow of their hearing system (Wever, 1978). The combined presence of previously unrecognized perilymphatic fenestrae and an expanded stapedial footplate indicate that the hearing system of *Proganochelys* may have already been a fully tympanic, essentially modern turtle re-entrant fluid flow system, contradicting previous interpretations based on the absence of these features (e.g., Clack & Allin, 2004; Foth et al., 2019; Gaffney, 1990; Sobral et al., 2016). Thus, the evolution of the re-entrant fluid flow system of the inner ear may have preceded morphological changes in the middle ear area, including the full formation of a cavum tympani as a funnel-shaped that compartmentalizes the turtle middle ear, the formation of a cavum acustico-jugulare and recessus scalae tympani, and the development of a ventrally projecting processus interfenestralis (e.g., Anquetin, 2010; Foth et al., 2019). The CT data of the Swiss specimen further revealed intraspecific variation in the carotid artery and some deviation of nerve foramina in the braincase in *Proganochelys quenstedtii*.

In the Frick specimen, the morphology of the articulated pes is very similar to that described for SMNS 17204 and SMNS 16980 (Gaffney, 1990). Distal tarsal 4 is also the largest element in the series, with distal tarsals being distinctly smaller; distal tarsal 1, however, is intermediate in size of the aforementioned elements and larger than proposed in the reconstructed pes (Gaffney, 1990: p. 251). Finally, we propose that sesamoid bones were not restricted to the forelimb autopod, but were also present in the hind feet of *Proganochelys quenstedtii*.

## Supplementary Information

**Additional file 1:** **Fig. S1** Recovered skull elements of *Proganochelys quenstedtii* (SMF 09-F2). **A,** Partially reassembled bones. **B,** Interpretative sketch and identification of the skull elements.

**Additional file 2:** **Fig. S2** Spatial comparison of the skull elements *Proganochelys quenstedtii* (SMF 09-F2) with newly generated 3D surface model of SMNS 16980 (not to scale; original CT scan data set from Werneburg et al., 2015 which was reused in Lautenschlager et al., 2018). Note that the right part of the SMNS 16980 skull was not labelled and included due to a break in the specimen. Scale bar only for SMF 09-F2 bones. **A,** Left prefrontal and left postorbital. **B,** Left maxilla. **C,** Left quadratojugal and fragment of left jugal. **D,** Left quadrate. **E,** Both frontals. **F,** Both nasals and potentially part of right prefrontal. Note that sutures are note visible. **G,** Right maxilla with left and right premaxilla and fragment of right jugal. **H,** Posterior skull portion. Again, sutures are note visible. Abbreviations: fm, foramen magnum; fr, frontal; ju, jugal; mx, maxilla; n, nasal; occ, occipital condyle; pmx, premaxilla; po, postorbital; pf, prefrontal; q, quadrate; qj, quadratojugal.

**Additional file 3:** **Fig. S3** Carapace of *Proganochelys quenstedtii* (SMF 09-F2) with interpretative drawings of scute sulci superimposed on the shell bones. **A,** Carapace in angled anterodorsolateral view. **B,** Right side of carapace in dorsolateral view. **C,** Right side of carapace in lateral view. Reconstructed parts of the carapace are delimited by grey-shaded area set off by a stippled white line and marked with a white r. The anterior aspect of the carapace is marked in each view by a black arrow. Abbreviations: c, cervical scute; m, marginal scute; pl, pleural scute; sc, supracaudal scute; sm, supramarginal scute; v, vertebral scute.

**Additional file 4:** **Fig. S4** Carapace of *Proganochelys quenstedtii* (SMF 09-F2) during preparation. Large white arrows indicate cranial direction. **A,** Internal/visceral view of carapace, with the dorsal side still resting on the plaster jacket. **B,** Close-up view of the anterior portion of the carapace with shallow grooves between costals being indicated by small white arrows.

**Additional file 5:** **Fig. S5** Right hind limb elements of *Proganochelys quenstedtii* (SMF 09-F2) during preparation. **A,** Matrix block with zeugopodial elements, limb osteoderms and few autopodial elements. **B,** Reverse side of matrix block showing most of the autopodium still in articulation.

## Data Availability

All data generated or analysed during this study are included in this published article and the fossil described herein is officially accessioned and available upon request at the Dinosauriermuseum Frick. The 3D models of the posterior skull portion and underlying CT scans are made available on Morphosource repository project ID: 000447389 (https://www.morphosource.org/projects/000447389).
