# Supplementary figures and images for: First evidence of Proganochelys quenstedtii (Testudinata) from the Plateosaurus bonebeds (Norian, Late Triassic) of Frick, Canton Aargau, Switzerland
Source: Swiss J Palaeontol. 2022 Oct 27;141(1):17. doi: 10.1186/s13358-022-00260-4 (PMC9613585; doi:10.1186/s13358-022-00260-4)

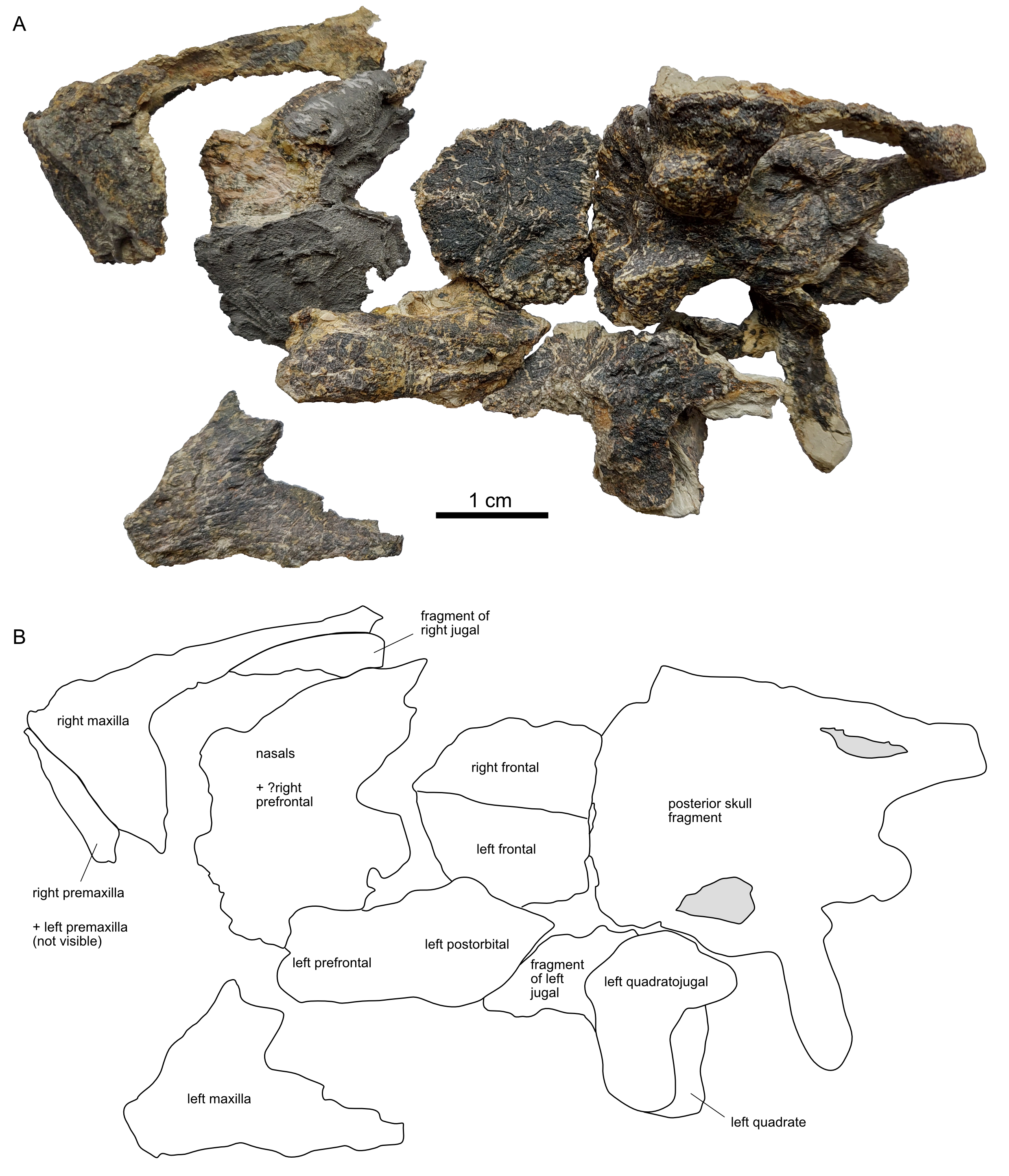

Supplement: Supplementary file 1 — Additional file 1: Fig. S1 Recovered skull elements of Proganochelys quenstedtii (SMF 09-F2). A, Partially reassembled bones. B, Interpretative sketch and identification of the skull elements. [file 13358_2022_260_MOESM1_ESM.tiff]

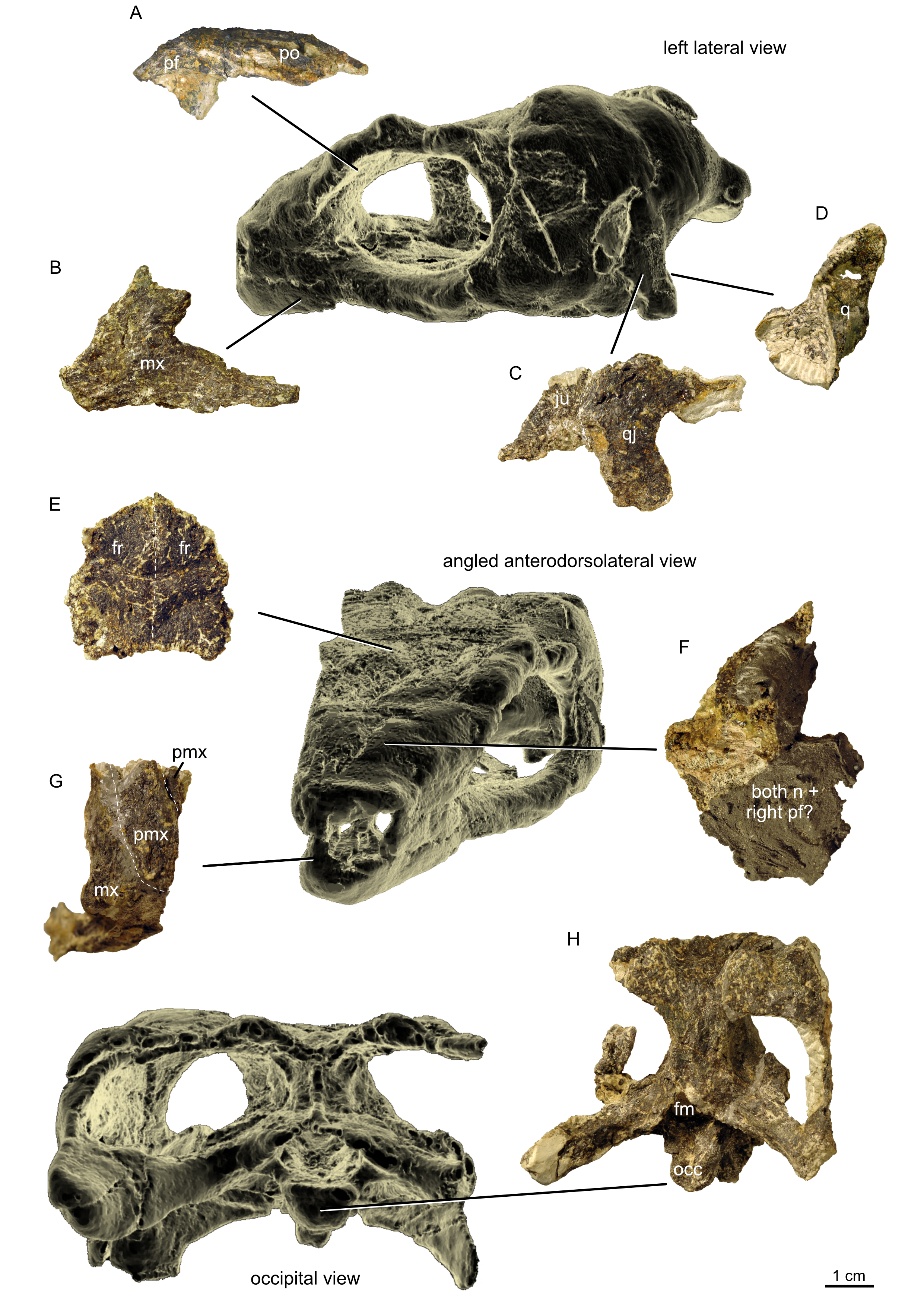

Supplement: Supplementary file 2 — Additional file 2: Fig. S2 Spatial comparison of the skull elements Proganochelys quenstedtii (SMF 09-F2) with newly generated 3D surface model of SMNS 16980 (not to scale; original CT scan data set from Werneburg et al., 2015 which was reused in Lautenschlager et al., 2018). Note that the right part of the SMNS 16980 skull was not labelled and included due to a break in the specimen. Scale bar only for SMF 09-F2 bones. A, Left prefrontal and left postorbital. B, Left maxilla. C, Left quadratojugal and fragment of left jugal. D, Left quadrate. E, Both frontals. F, Both nasals and potentially part of right prefrontal. Note that sutures are note visible. G, Right maxilla with left and right premaxilla and fragment of right jugal. H, Posterior skull portion. Again, sutures are note visible. Abbreviations: fm, foramen magnum; fr, frontal; ju, jugal; mx, maxilla; n, nasal; occ, occipital condyle; pmx, premaxilla; po, postorbital; pf, prefrontal; q, quadrate; qj, quadratojugal. [file 13358_2022_260_MOESM2_ESM.tiff]

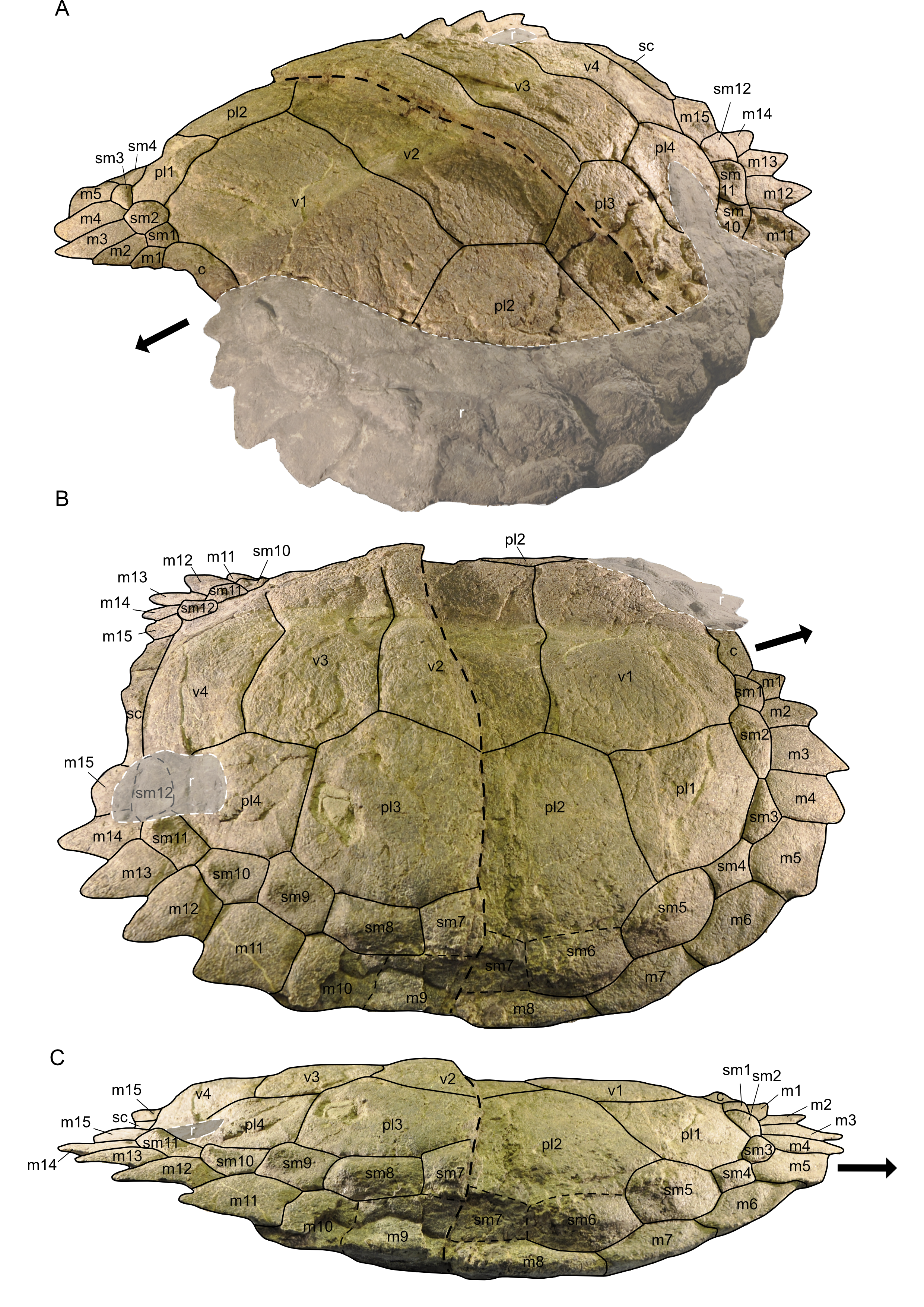

Supplement: Supplementary file 3 — Additional file 3: Fig. S3 Carapace of Proganochelys quenstedtii (SMF 09-F2) with interpretative drawings of scute sulci superimposed on the shell bones. A, Carapace in angled anterodorsolateral view. B, Right side of carapace in dorsolateral view. C, Right side of carapace in lateral view. Reconstructed parts of the carapace are delimited by grey-shaded area set off by a stippled white line and marked with a white r. The anterior aspect of the carapace is marked in each view by a black arrow. Abbreviations: c, cervical scute; m, marginal scute; pl, pleural scute; sc, supracaudal scute; sm, supramarginal scute; v, vertebral scute. [file 13358_2022_260_MOESM3_ESM.tiff]

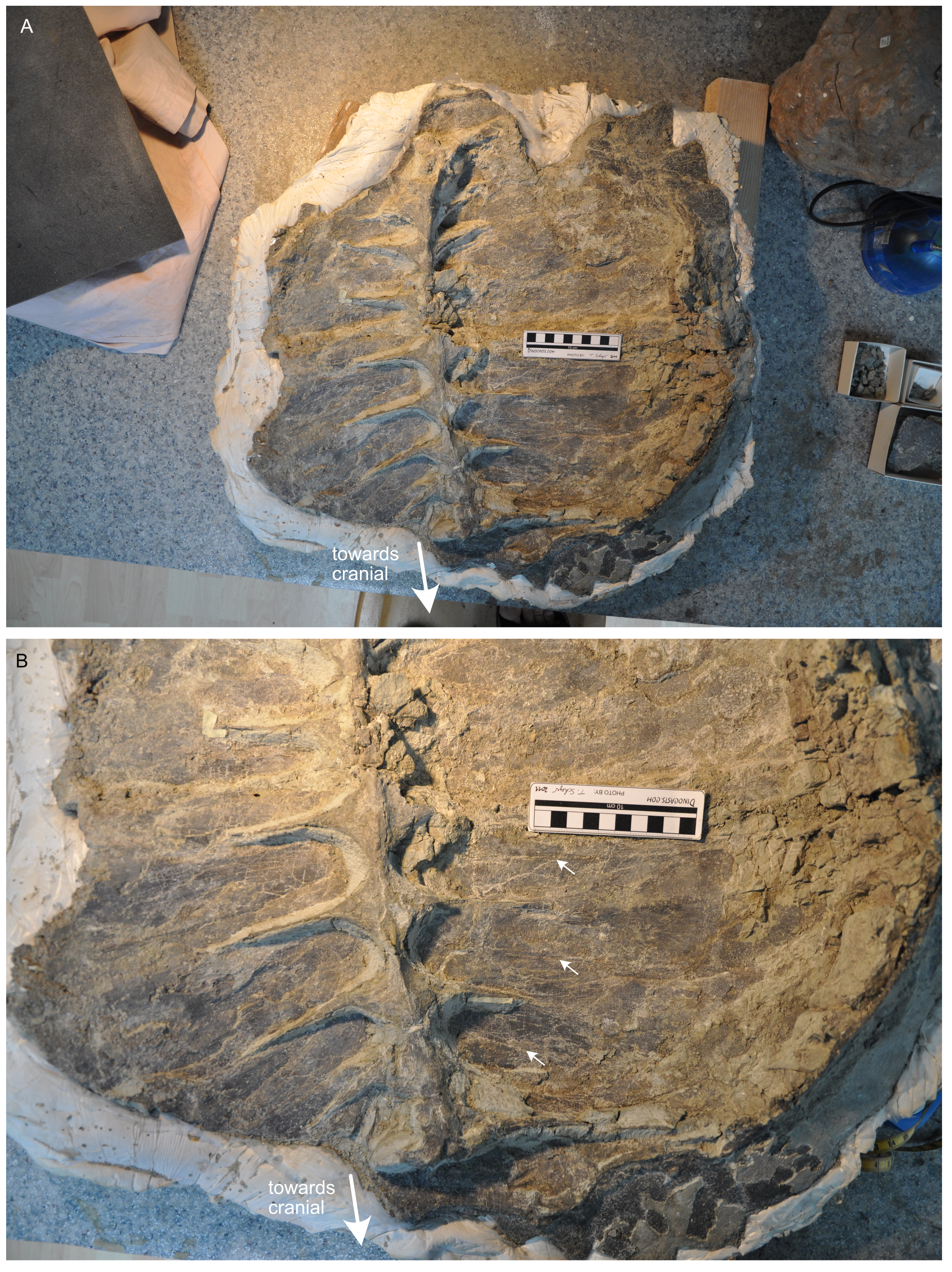

Supplement: Supplementary file 4 — Additional file 4: Fig. S4 Carapace of Proganochelys quenstedtii (SMF 09-F2) during preparation. Large white arrows indicate cranial direction. A, Internal/visceral view of carapace, with the dorsal side still resting on the plaster jacket. B, Close-up view of the anterior portion of the carapace with shallow grooves between costals being indicated by small white arrows. [file 13358_2022_260_MOESM4_ESM.tiff]

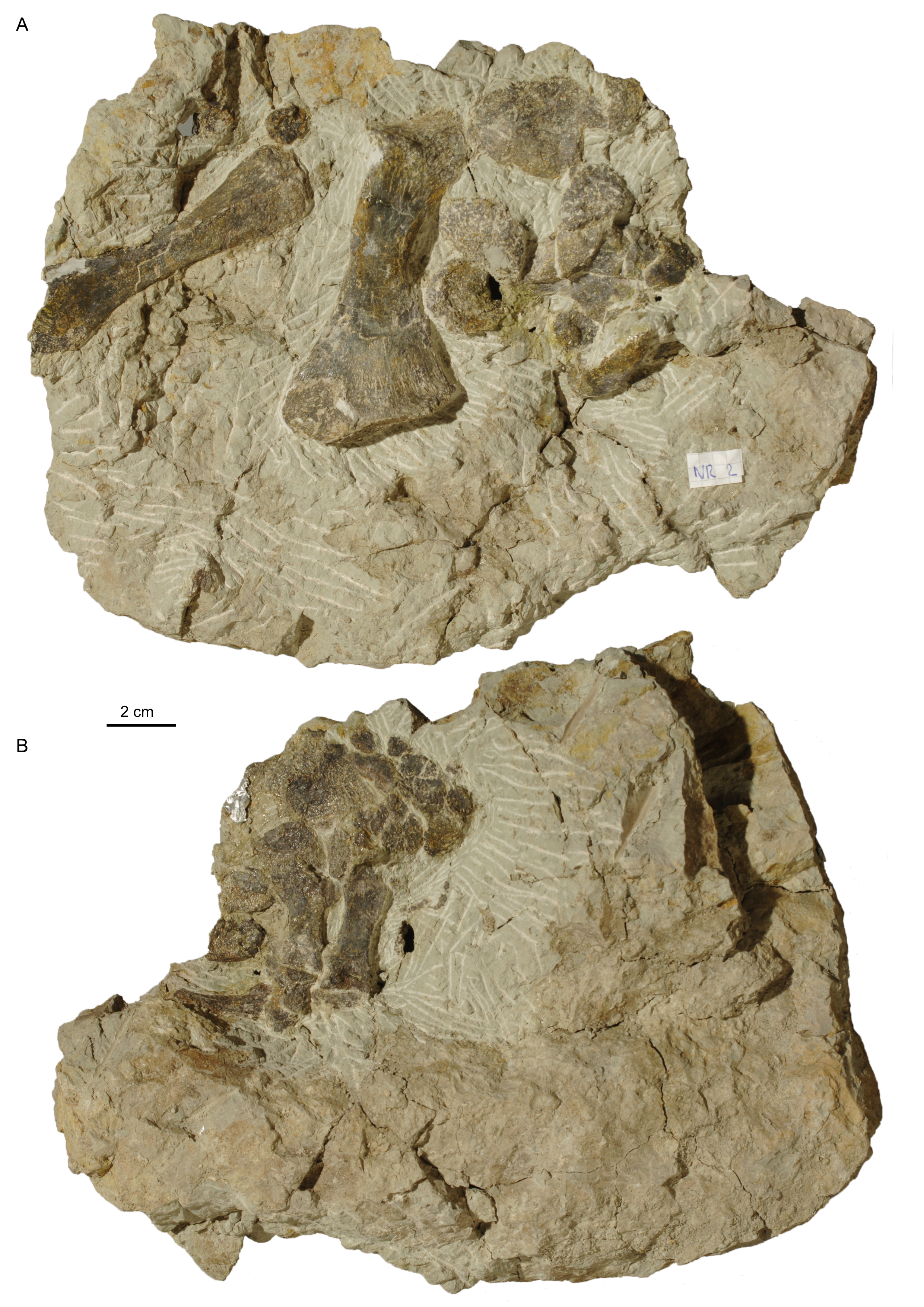

Supplement: Supplementary file 5 — Additional file 5: Fig. S5 Right hind limb elements of Proganochelys quenstedtii (SMF 09-F2) during preparation. A, Matrix block with zeugopodial elements, limb osteoderms and few autopodial elements. B, Reverse side of matrix block showing most of the autopodium still in articulation. [file 13358_2022_260_MOESM5_ESM.tiff]
